# Antibiotic Use, Bacterial Co-Infection, and Antimicrobial Resistance in Adults Hospitalized with COVID-19, Influenza, or RSV: A Systematic Review and Meta-Analysis

**DOI:** 10.3390/antibiotics15070654

**Published:** 2026-06-30

**Authors:** Florina Cristiana Lucaciu, Ovidiu Rosca, Ana Maria Mihai, Alexandra Sima, Madalina-Ianca Suba, Norbert Wellmann, Alessia Rosian, Cristian Oancea, Monica Cialma, Andrada Tarau, Alexandra Bosoanca, Monica Marc

**Affiliations:** 1 Doctoral School, “Victor Babes” University of Medicine and Pharmacy, 2 Eftimie Murgu Square, 300041 Timisoara, Romania; florina.lucaciu@umft.ro (F.C.L.); norbert.wellmann@umft.ro (N.W.); alessia.rosian@umft.ro (A.R.); andrada.dulf@umft.ro (A.T.); alexandra.bosoanca@umft.ro (A.B.); 2 Clinical Hospital of Infectious Diseases and Pulmonology “Victor Babes”, Gheorghe Adam Street 13, 300310 Timisoara, Romania; madalina.suba@umft.ro (M.-I.S.); oancea@umft.ro (C.O.); cialma.monica@gmail.com (M.C.); marc.monica@umft.ro (M.M.); 3 Department XIII, Discipline of Infectious Diseases, “Victor Babes” University of Medicine and Pharmacy, Eftimie Murgu Square 2, 300041 Timisoara, Romania; 4 Department of Diabetes, “Pius Brinzeu” Emergency Hospital, 300723 Timisoara, Romania; sima.alexandra@umft.ro; 5 Second Department of Internal Medicine, Faculty of Medicine, “Victor Babes” University of Medicine and Pharmacy, 300041 Timisoara, Romania; 6 Center of Research and Innovation in Personalized Medicine of Respiratory Diseases (CRIPMRD), “Victor Babes” University of Medicine and Pharmacy, 2 Eftimie Murgu Square, 300041 Timisoara, Romania

**Keywords:** antimicrobial stewardship, empirical antibiotic prescribing, secondary bacterial infection, multidrug-resistant organisms, viral respiratory infection, prescribing-to-confirmation gap, SARS-CoV-2, respiratory syncytial virus

## Abstract

Background: Adults hospitalized with COVID-19, influenza A/B, or respiratory syncytial virus (RSV) frequently receive empirical antibiotics, but antibiotic prescribing, confirmed bacterial co-infection, antimicrobial resistance (AMR), and outcomes have not been jointly synthesized across these infections. Methods: We conducted a PRISMA 2020 systematic review and meta-analysis of 39 studies including 839,531 hospitalized adults. Random-effects models with Freeman–Tukey double-arcsine transformation pooled prevalence estimates; sensitivity and publication-bias analyses were performed where appropriate. Results: Pooled antibiotic use was 62.56% (95% CI, 53.75–70.97%) for COVID-19, 57.48% (25.76–86.09%) for influenza A/B, and 76.03% (67.62–83.53%) for RSV, with very high heterogeneity. Confirmed bacterial co-infection was lower: 5.31% (3.43–7.56%), 18.66% (9.98–29.30%), and 24.36% (18.53–30.70%), respectively. Prescribing-to-confirmed infection ratios ranged from 3.0 to 46.2. AMR evidence was restricted to COVID-19 studies and was dominated by carbapenem-resistant Gram-negative organisms, mainly in secondary, ICU-associated, or healthcare-associated infections. Confirmed bacterial complications were associated with ICU admission, longer hospitalization, and higher mortality. Conclusions: Antibiotic prescribing exceeded confirmed bacterial infection across all viral groups, but estimates require cautious interpretation due to heterogeneity, diagnostic uncertainty, observational evidence, and the absence of low-risk-of-bias studies. The evidence base was dominated by COVID-19 cohorts, while influenza A/B and RSV data, especially virus-specific AMR evidence, remain limited. COVID-19-specific AMR findings should not be generalized to influenza A/B or RSV. Virus-specific stewardship should prioritize rapid diagnostics, systematic sampling, reassessment, and de-escalation when bacterial infection is not confirmed.

## 1. Introduction

Acute respiratory infections caused by influenza A/B viruses, respiratory syncytial virus (RSV), and severe acute respiratory syndrome coronavirus 2 (SARS-CoV-2) continue to represent major causes of hospitalization, clinically apparent acute respiratory failure, and death among adults, particularly older patients and those with pre-existing chronic conditions [1]. Although the clinical impact of coronavirus disease 2019 (COVID-19) has changed compared with the beginning of the pandemic, these three viruses continue to circulate concurrently, especially during the cold season, creating diagnostic and therapeutic challenges in acute-care settings. RSV, previously considered a predominantly pediatric pathogen, is now increasingly recognized as an important cause of severe lower respiratory tract disease, particularly among older adults and medically vulnerable individuals. In certain groups, hospitalization and mortality rates have been observed to be comparable to those associated with influenza A/B [1,2].

Among adults hospitalized with viral respiratory infections, an important clinical challenge lies in distinguishing primary viral disease from bacterial complications. Bacterial co-infections present at the time of admission or developing shortly thereafter, as well as secondary bacterial infections or superinfections that arise later during hospitalization, represent distinct entities with different prognostic and therapeutic implications. Damage to the respiratory epithelium caused by viral infection, impaired mucociliary clearance, and immune response dysfunction facilitate bacterial invasion, which may lead to pneumonia, sepsis, or even septic shock. Nevertheless, the similarity between the clinical manifestations of severe viral infections and those of bacterial infections often makes early decisions regarding the initiation of empirical antibiotic therapy difficult [3].

In COVID-19, this diagnostic uncertainty is particularly evident. Existing systematic reviews have shown that bacterial co-infection at presentation was uncommon, whereas antibiotics were prescribed to the majority of patients hospitalized with viral infection, a discrepancy subsequently confirmed in several meta-analyses [3,4,5]. Bacterial complications are also clinically significant in influenza, which is particularly associated with secondary pneumonia caused by Streptococcus pneumoniae, Staphylococcus aureus, and Haemophilus influenzae. A recent meta-analysis estimated that bacterial infection occurs in approximately 11.2% of patients hospitalized with influenza, increasing the risk of mortality by approximately 3.4-fold [6]. In adults hospitalized with RSV, the evidence is more limited but is growing, with observational data reporting the presence of bacterial co-infection, frequent use of empirical antibiotic therapy, and unfavorable clinical outcomes among patients with bacterial complications [7,8].

The consequences of the discrepancy between antibiotic prescribing and the confirmation of bacterial infection extend beyond the individual patient. Global studies indicate that bacterial antimicrobial resistance (AMR) is responsible for a significant proportion of mortality caused by infectious diseases, with lower respiratory tract infections representing one of the major contributors to AMR-associated deaths [9]. Among patients hospitalized with COVID-19, particularly those who are critically ill, AMR is especially common in patients who develop secondary healthcare-associated infections [10]. The increasingly frequent initiation of empirical antibiotic therapy, even in the absence of confirmed bacterial infection, promotes the emergence of resistant microorganisms, disrupts the microbiome, and generates potentially avoidable complications. This highlights the direct relationship between prescribing practices and the emergence of AMR in hospital settings. The risks are even greater among patients with pre-existing chronic respiratory conditions, such as chronic obstructive pulmonary disease (COPD) or asthma, who account for a significant proportion of patients hospitalized with RSV, influenza A/B, and COVID-19. These patients are at increased risk of severe disease progression and are more likely to receive empirical antibiotics, thereby constituting an important subgroup for the implementation of targeted antimicrobial stewardship strategies [11,12].

Although existing systematic reviews have separately examined bacterial co-infection or antibiotic prescribing in COVID-19, as well as bacterial complications in influenza A/B and RSV, we identified no study that directly compared antibiotic use, confirmed bacterial co-infection, AMR patterns, and associated clinical outcomes across all three major viral respiratory infections within a unified framework. This gap is clinically relevant because adults hospitalized with COVID-19, influenza A/B, or RSV may initially present with similar clinical syndromes. However, the frequency, timing, and prognostic significance of bacterial complications may vary considerably according to the identified virus, with direct implications for antimicrobial stewardship strategies.

In this systematic review and meta-analysis, we aim to estimate the pooled prevalence of reported antibiotic use and confirmed bacterial co-infection among adults hospitalized with COVID-19, influenza A/B, or RSV; to descriptively characterize the differences between these two outcomes within each viral infection group and across groups; to synthesize the reported patterns of antimicrobial resistance (AMR); and to evaluate the available evidence regarding clinical outcomes associated with antibiotic exposure, bacterial co-infection, secondary infection, or multidrug-resistant infection. These findings are intended to support diagnostic reassessment and the development of virus-specific antimicrobial stewardship strategies in hospitalized adult populations, including patients with chronic respiratory diseases.

## 2. Results

### 2.1. Study Selection

**Figure 1:**
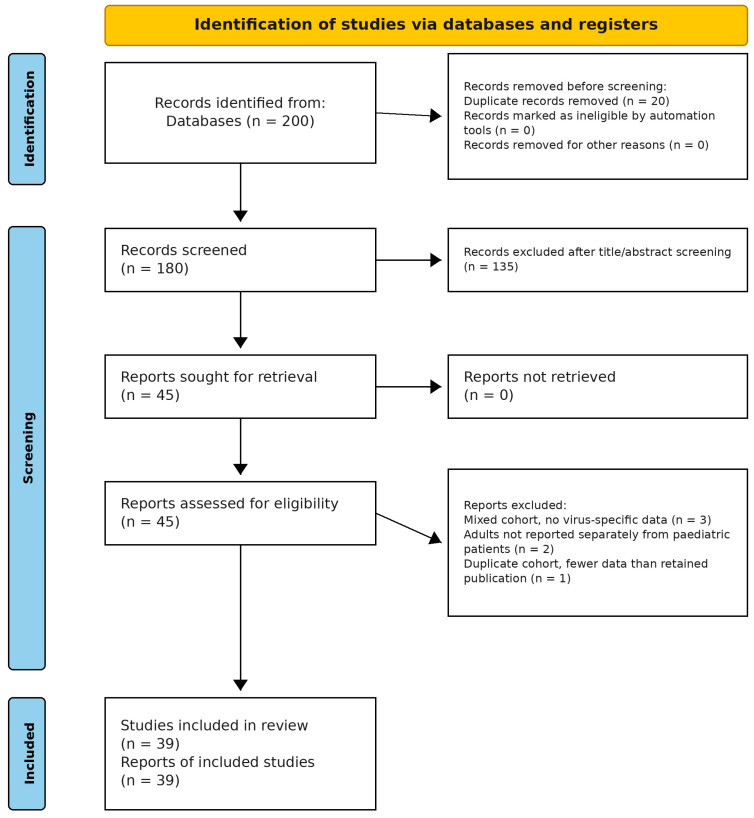
PRISMA 2020 flow diagram for the identification, screening, eligibility assessment, and inclusion of studies in the systematic review and meta-analysis. Arrows indicate the flow of records through each stage of the selection process, while the colored sections indicate the corresponding PRISMA phases.

For this study, the systematic search identified 200 records from electronic databases (PubMed/MEDLINE, Embase, Cochrane CENTRAL, and Web of Science). Twenty records were removed as duplicates, and 180 unique records underwent title and abstract screening, of which 135 were excluded because they did not meet the predefined eligibility criteria. Full texts were obtained for the remaining 45 records. Following full-text assessment, six reports were excluded: three reported mixed cohorts of respiratory viruses without extractable virus-specific data for individual pathogens; two did not report adult data separately from pediatric patients; and one was identified as a duplicate cohort report that provided fewer outcomes than a retained publication from the same study population. Ultimately, 39 studies were included in the systematic review and final meta-analysis. The study selection process is presented in Figure 1.

Of the 39 included studies, 33 contributed data to at least one quantitative meta-analysis. More specifically, 19 studies contributed to the quantitative synthesis of reported antibiotic use (Section 2.4), while 17 studies contributed to the quantitative synthesis of confirmed bacterial co-infection (Section 2.5); three studies contributed to both syntheses. Five studies contributed to the within-cohort descriptive comparison between reported antibiotic use and confirmed bacterial co-infection in the same patient population (Section 2.6). Eight studies provided direct virus-specific AMR data from COVID-19 cohorts (Section 2.7), while two additional studies provided contextual AMR evidence from mixed viral respiratory cohorts in which findings could not be attributed separately to influenza A/B or RSV. These contextual studies were not used to infer virus-specific AMR patterns for influenza A/B or RSV. Fourteen studies provided data for the narrative synthesis of clinical outcomes (Section 2.8). Several studies contributed to more than one synthesis; study-level contributions are detailed in Table 1.

### 2.2. Characteristics of the Included Studies

The main characteristics of the 39 included studies are summarized in Table 1; full study-level details, including study design, study period, and clinical setting, are provided in Supplementary Table S1. The studies were published between 2014 and 2026, with the vast majority (*n* = 31) reporting data on adults hospitalized with COVID-19, reflecting the volume of research generated during and following the SARS-CoV-2 pandemic. Ten studies included patients hospitalized with influenza A or B, and eight included patients with RSV infection; these categories were not mutually exclusive, as seven studies reported data on more than one eligible viral respiratory infection.

All included studies had an observational design. The studies were conducted across several geographical regions, including Europe, North America, South America, Asia, the Middle East, and Africa. Most studies included adults admitted to general hospital wards or to both general ward and intensive care unit (ICU) settings; four studies focused exclusively on ICU or critically ill populations. For the purpose of descriptive interpretation, clinical settings were categorized as general ward/non-ICU, mixed ward/ICU, ICU/critically ill, or unclear/not separately reported. Because most studies did not provide outcome data stratified separately for ICU and non-ICU patients, no formal meta-analysis by clinical setting was performed. Instead, ICU versus non-ICU status was considered descriptively in the within-cohort comparison, AMR synthesis, clinical outcome synthesis, and interpretation of generalizability. Sample sizes varied considerably, ranging from 9 to 620,630 participants, reflecting differences in study design and the inclusion of large administrative or registry-based cohorts alongside smaller, single-center studies.

### 2.3. Risk of Bias Assessment

The risk of bias was assessed for all 39 included studies using critical appraisal tools appropriate for each study design, in accordance with the Joanna Briggs Institute (JBI) methodology. More specifically, the JBI Checklist for Cohort Studies was applied to cohort studies; the JBI Checklist for Analytical Cross-Sectional Studies was used for analytical cross-sectional studies and studies based on administrative databases; and the relevant JBI checklists were applied to case series and, respectively, prevalence studies based on laboratory data. Of these studies, 26 were considered to present moderate methodological concerns, while 13 raised major methodological concerns; no study was considered to have a low risk of bias. Details of the risk-of-bias assessment for each individual study are presented in Supplementary Table S2.

The most frequently identified limitations were related to the retrospective design of the studies, microbiological testing performed selectively at the clinician’s discretion or without uniform standardization, variations in the definition and timing of antibiotic exposure or bacterial co-infection, as well as residual confounding in analyses of clinical outcomes. Studies that investigated associations between antibiotic exposure and unfavorable outcomes were particularly susceptible to confounding, because antibacterial treatment was frequently initiated in patients with greater disease severity or suspected bacterial infection. Studies limited to intensive care unit (ICU) populations, highly selected microbiologically positive cohorts, or case series provided clinically relevant evidence regarding secondary infections and antimicrobial resistance; however, their generalizability to the broader population of hospitalized adults was limited. These limitations were taken into account when interpreting all pooled estimates and narrative syntheses reported in the following sections.

### 2.4. Reported Antibiotic Use

Antibiotics were reported as being used in 21 virus-specific cohorts derived from 19 studies, including a total of 823,874 adults hospitalized with COVID-19, influenza A or B, or RSV infection [13,14,15,16,17,18,19,20,21,22,23,24,25,26,27,28,29,30]. Two studies contributed independent cohorts for more than one eligible viral infection: Karlsen et al. reported separate influenza and RSV cohorts [26], while Homen Fernandez et al. reported separate COVID-19 and RSV cohorts [29]. Using a random-effects model, the pooled prevalence of reported antibiotic use across all viral cohorts was 65.18% (95% CI, 58.26–71.79%), with considerable between-study heterogeneity (I^2^ = 99.96%; *p* < 0.0001) (Table 2).

**Figure 2:**
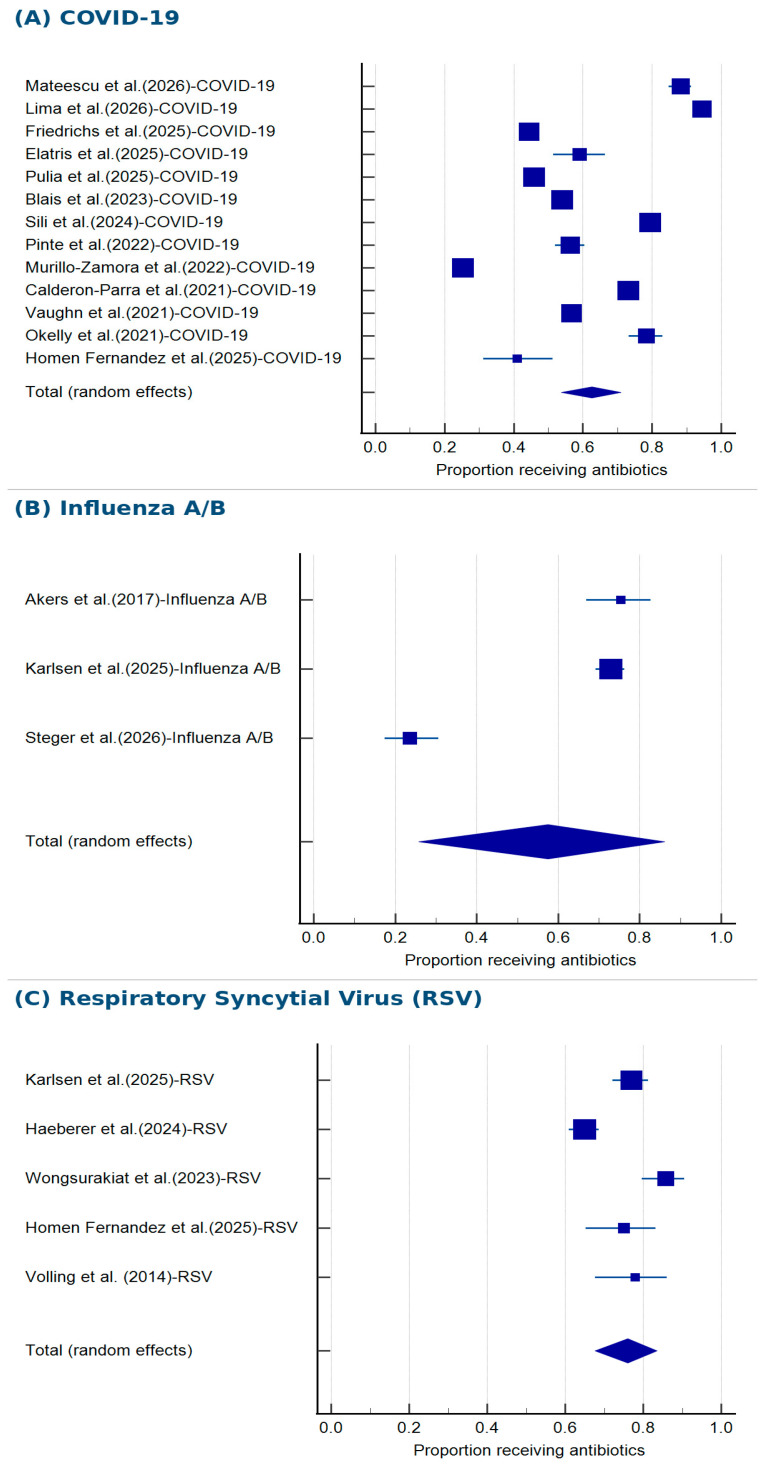
Forest plots of reported antibiotic use among adults hospitalized with COVID-19, influenza, or respiratory syncytial virus infection. Panel (**A**) presents the random-effects pooled prevalence of reported antibiotic use among adults hospitalized with COVID-19; Panel (**B**) presents the corresponding pooled estimate among adults hospitalized with influenza; and Panel (**C**) presents the pooled estimate among adults hospitalized with RSV infection. Squares represent study-specific prevalence estimates, with the size of each square proportional to the weight of the corresponding study in the meta-analysis. Horizontal lines represent 95% confidence intervals, and diamonds represent random-effects pooled estimates, with the width of each diamond indicating the corresponding 95% confidence interval [7,13,14,15,16,17,18,19,20,21,22,23,24,25,26,27,28,29,30].

Given the very high between-study heterogeneity, the pooled estimates of antibiotic use should be interpreted with caution. These estimates should not be considered precise prevalence values applicable to all hospitalized adults with viral respiratory infections, but rather descriptive summary measures across clinically and methodologically diverse cohorts. For this reason, virus-specific pooled estimates were considered more clinically informative than the overall estimate and are presented separately below (Table 2; Figure 2).

Among adults hospitalized with COVID-19, 13 cohorts comprising 821,592 patients contributed to the quantitative synthesis [13,14,15,16,17,18,19,20,21,22,23,24,29]. The random-effects pooled prevalence of reported antibiotic use was 62.56% (95% CI, 53.75–70.97%), with considerable between-study heterogeneity (I^2^ = 99.97%; *p* < 0.0001) (Table 2; Figure 2A). Study-specific estimates ranged from approximately 25% (Murillo-Zamora et al. [21]) to 94% (Lima et al. [14]), consistent with substantial variation in patient populations, clinical settings, pandemic periods, and operational definitions of antibiotic exposure.

For influenza A or B, three cohorts comprising 934 hospitalized adults provided data [25,26,27]. The random-effects pooled prevalence of reported antibiotic use was 57.48% (95% CI, 25.76–86.09%), with considerable heterogeneity (I^2^ = 98.70%; *p* < 0.0001) (Table 2; Figure 2B). The wide confidence interval reflects the limited number of included cohorts and the marked variability across studies; therefore, this estimate should be interpreted with caution.

For RSV infection, five cohorts comprising 1348 hospitalized adults were available for quantitative synthesis [7,26,28,29,30]. The random-effects pooled prevalence of reported antibiotic use was 76.03% (95% CI, 67.62–83.53%), with considerable heterogeneity (I^2^ = 90.20%; *p* < 0.0001) (Table 2; Figure 2C).

Reported antibiotic use was frequent among adults hospitalized with each of the three viral respiratory infections, with pooled estimates exceeding 57% in all viral subgroups. The highest point estimate was observed in the RSV subgroup; however, differences between groups should be interpreted descriptively rather than as formal comparative effects, given the substantial heterogeneity and the considerably different sample sizes across subgroups.

Sensitivity analysis: A prespecified sensitivity analysis restricted to studies assessed as having moderate methodological concerns was performed to evaluate the robustness of the primary estimates. In the COVID-19 subgroup, exclusion of one study with high methodological concerns (O’Kelly et al. [24]) yielded a pooled prevalence of 61.17% (95% CI, 51.98–69.98%; I^2^ = 99.98%), consistent with the primary estimate and confirming the robustness of the main finding. For the influenza subgroup, sensitivity analysis was not feasible because, after excluding studies with high methodological concerns, only one cohort with moderate methodological concerns remained [25,27]; the individual estimate for the retained study was 72.87% (Karlsen et al. [26]). In the RSV subgroup, exclusion of the only study with high methodological concerns (Haeberer et al. [28]) yielded a pooled prevalence of 79.07% (95% CI, 73.90–83.80%; I^2^ = 58.20%) across four cohorts, with a notable reduction in heterogeneity compared with the primary estimate, suggesting that Haeberer et al. contributed substantially to between-study variability in this subgroup. The forest plot for the COVID-19 antibiotic use sensitivity analysis is presented in Supplementary Figure S1.

No formal assessment of publication bias was performed for the influenza and RSV subgroups because the number of included cohorts, three and five, respectively, was below the recommended minimum threshold for a meaningful analysis. For the COVID-19 subgroup (13 cohorts), Egger’s test did not demonstrate statistically significant asymmetry (intercept = 22.08; 95% CI, −23.66 to 67.81; *p* = 0.3108), and Begg’s test was similarly non-significant (Kendall’s tau = −0.41; *p* = 0.0509), providing no evidence of publication bias.

### 2.5. Confirmed Bacterial Co-Infection

**Figure 3:**
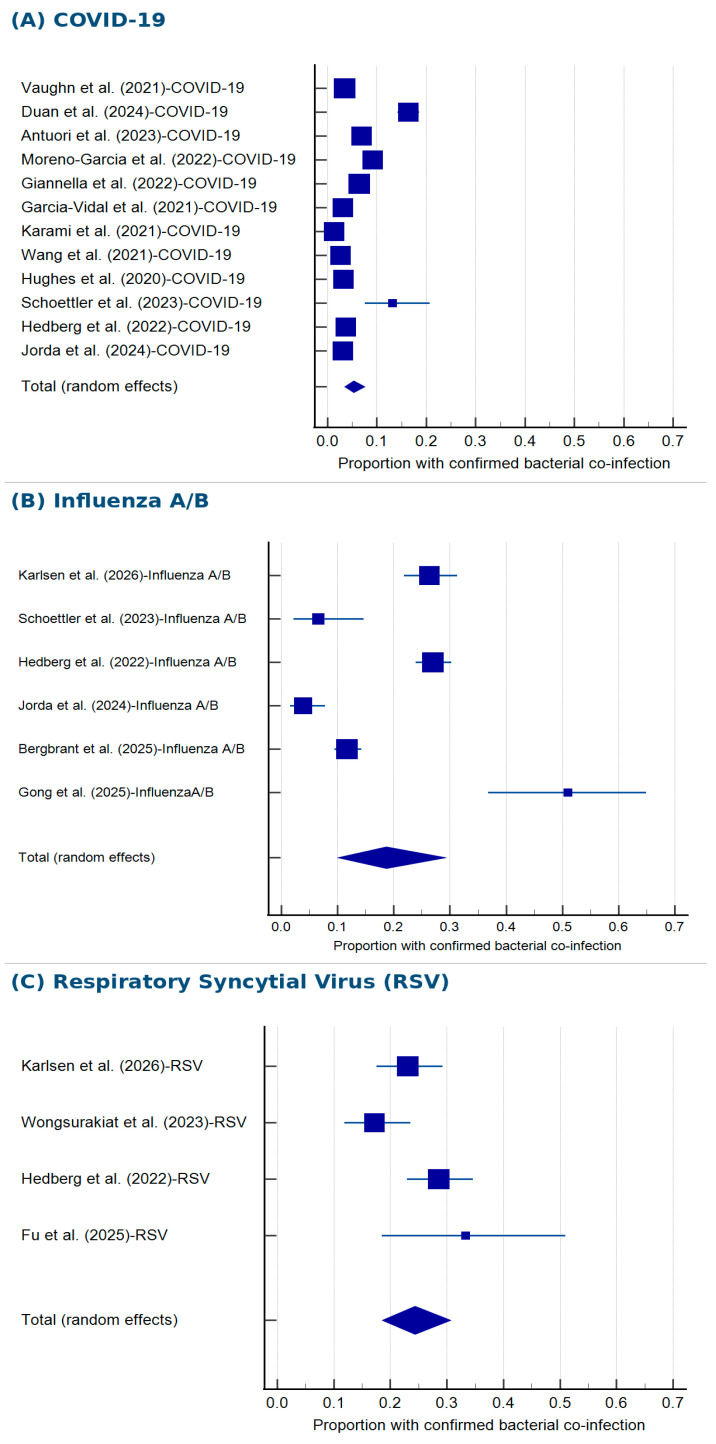
Forest plots of the random-effects pooled prevalence of confirmed bacterial co-infection among adults hospitalized with COVID-19 (Panel (**A**)), influenza A or B (Panel (**B**)), or respiratory syncytial virus infection (Panel (**C**)). Squares represent study-specific prevalence estimates, with the size of each square proportional to the weight of the corresponding study in the meta-analysis. Horizontal lines represent 95% confidence intervals, and diamonds represent random-effects pooled estimates, with the width of each diamond indicating the corresponding 95% confidence interval [7,23,26,31,32,33,34,35,36,37,38,39,40,41,42,43,44].

Data on confirmed bacterial co-infection were identified in 22 virus-specific cohorts derived from 17 studies, comprising a total of 16,309 adults hospitalized with COVID-19, influenza A or B, or RSV infection [23,26,29,31,32,33,34,35,36,37,38,39,40,41]. Four studies contributed independent cohorts for more than one eligible viral infection: Karlsen et al. reported separate influenza and RSV cohorts [26]; Schoettler et al. reported separate COVID-19 and influenza cohorts [39]; Hedberg et al. reported separate COVID-19, influenza, and RSV cohorts [40]; and Jorda et al. reported separate COVID-19 and influenza cohorts [41]. Across all eligible viral cohorts, the random-effects pooled prevalence of confirmed bacterial co-infection was 11.04% (95% CI, 7.83–14.72%), with considerable between-study heterogeneity (I^2^ = 97.93%; *p* < 0.0001). Given the very high between-study heterogeneity, the pooled estimates of confirmed bacterial co-infection should be interpreted with caution. These estimates summarize data from cohorts that differed substantially in clinical setting, illness severity, microbiological sampling strategy, timing of infection assessment, and definitions of bacterial co-infection. Therefore, the pooled estimates should be viewed as descriptive indicators of the available evidence rather than precise or universally generalizable prevalence values (Table 3; Figure 3).

In this quantitative synthesis, confirmed bacterial co-infection refers primarily to microbiologically confirmed bacterial infection reported at admission or within the first 48 h of hospitalization, according to the definitions used by the original studies. When studies reported secondary infection or superinfection separately, these later-onset infections were not combined with admission co-infection estimates unless the original study reported a composite bacterial infection outcome that could not be disaggregated. Studies with unclear timing or mixed definitions were retained as reported and interpreted cautiously. Therefore, pooled estimates should be understood as estimates of confirmed bacterial co-infection as defined by the contributing studies, rather than as a uniform measure of all bacterial complications occurring during hospitalization.

Among adults hospitalized with COVID-19, 12 cohorts comprising 13,471 patients contributed to the quantitative synthesis [23,31,32,33,34,35,36,37,38,39,40,41]. The random-effects pooled prevalence of confirmed bacterial co-infection was 5.31% (95% CI, 3.43–7.56%), with considerable between-study heterogeneity (I^2^ = 96.54%; *p* < 0.0001) (Table 3; Figure 3A).

For influenza A or B, six cohorts comprising 2168 hospitalized adults were included in the meta-analysis [26,39,40,41,42,43]. The random-effects pooled prevalence of confirmed bacterial co-infection was 18.66% (95% CI, 9.98–29.30%), with considerable heterogeneity (I^2^ = 96.64%; *p* < 0.0001) (Table 3; Figure 3B). The wide confidence interval reflects both the limited number of contributing cohorts and the substantial between-study variability.

For RSV infection, four cohorts comprising 670 hospitalized adults were available for quantitative synthesis [26,29,40,44]. The random-effects pooled prevalence of confirmed bacterial co-infection was 24.36% (95% CI, 18.53–30.70%), with substantial heterogeneity (I^2^ = 67.68%; *p* = 0.0258), although heterogeneity was lower than that observed in the COVID-19 and influenza subgroups (Table 3; Figure 3C).

Confirmed bacterial co-infection was identified in a minority of hospitalized adults across all three viral infection groups. The pooled point estimate was numerically lowest among patients hospitalized with COVID-19 and substantially higher among those with influenza or RSV, suggesting clinically relevant between-virus differences in the frequency of confirmed bacterial complications. These differences should be interpreted in the context of considerable heterogeneity and the smaller number of influenza A/B and RSV cohorts.

Sensitivity analysis: A prespecified sensitivity analysis restricted to studies assessed as having moderate methodological concerns confirmed the robustness of the primary estimates across all three viral subgroups. In the COVID-19 subgroup, exclusion of two studies with high methodological concerns (Moreno-García et al. [33] and Schoettler et al. [39]) yielded a pooled prevalence of 4.51% (95% CI, 2.70–6.76%; I^2^ = 96.71%) across 10 cohorts, consistent with the primary estimate. In the influenza A/B subgroup, exclusion of two studies with high methodological concerns (Schoettler et al. [39] and Gong et al. [43]) yielded a pooled prevalence of 16.06% (95% CI, 7.27–27.46%; I^2^ = 97.34%) across four cohorts, likewise consistent with the primary estimate. In the RSV subgroup, exclusion of one study with high methodological concerns (Fu et al. [44]) yielded a pooled prevalence of 23.03% (95% CI, 17.02–29.66%; I^2^ = 73.15%) across three cohorts, with heterogeneity remaining substantial despite the exclusion. The forest plot for the COVID-19 bacterial co-infection sensitivity analysis is presented in Supplementary Figure S2.

Formal assessment of publication bias was not performed for the influenza A/B and RSV subgroups because of the insufficient number of studies. For the COVID-19 subgroup (12 cohorts), Egger’s test did not demonstrate statistically significant asymmetry (intercept = 3.23; 95% CI, −12.64 to 19.09; *p* = 0.6600), and Begg’s test was similarly non-significant (Kendall’s tau = 0.14; *p* = 0.5340), providing no evidence of publication bias.

### 2.6. Within-Cohort Descriptive Comparison Between Reported Antibiotic Use and Confirmed Bacterial Co-Infection

Five studies contributed seven virus-specific cohorts to a within-cohort descriptive comparison of reported antibiotic use and confirmed bacterial co-infection in the same patient population [7,23,32,36,40]. These cohorts are summarized in Table 4.

Among adults hospitalized with COVID-19, four cohorts provided data for this analysis [23,32,36,40]. Reported antibiotic use ranged from 33% to 60.1%, whereas confirmed bacterial co-infection was identified in 1.3% to 6.9% of patients within the same cohorts. The absolute difference between reported antibiotic use and confirmed bacterial co-infection ranged from 29 to 58.8 percentage points across the four cohorts. Antibiotics were prescribed at rates 6.1 to 46.2 times higher than the rate of confirmed bacterial co-infection, with the greatest discrepancy observed in the cohort reported by Karami et al. [36], in which confirmed bacterial co-infection was documented in only 1.3% of patients despite an antibiotic use rate of 60.1%.

One study including patients with influenza A or B provided within-cohort data (Hedberg et al. [40]). Reported antibiotic use was 84%, whereas confirmed bacterial co-infection was identified in 27% of patients, resulting in an absolute difference of 57 percentage points and a prescribing-to-infection ratio of 3.1.

Two cohorts of patients with RSV provided data for this analysis [7,40]. Reported antibiotic use was 85.7% and 88%, respectively, whereas confirmed bacterial co-infection was identified in 17.1% and 29% of patients, respectively, resulting in absolute differences of 68.6 and 59 percentage points and prescribing-to-infection ratios of 5.0 and 3.0, respectively.

When interpreted according to clinical setting, the prescribing-to-confirmation gap was not restricted to ICU-enriched cohorts. In COVID-19, both general ward cohorts and mixed ward/ICU cohorts showed consistently higher antibiotic use than confirmed bacterial co-infection. The largest prescribing-to-infection ratio was observed in a general ward COVID-19 cohort, indicating that empirical antibiotic overuse was not exclusively driven by ICU populations. Similarly, in RSV, high antibiotic use relative to confirmed bacterial co-infection was observed in both a general ward cohort and a mixed ward/ICU cohort. These findings suggest that the discrepancy between antibiotic prescribing and microbiologically confirmed bacterial infection extends across inpatient settings, although ICU-specific estimates could not be formally pooled because most studies did not report stratified ICU versus non-ICU data.

Across all seven virus-specific cohorts, reported antibiotic use consistently exceeded the prevalence of confirmed bacterial co-infection, with absolute differences ranging from 29 to 68.6 percentage points and prescribing-to-infection ratios ranging from 3.0 to 46.2. The greatest relative discrepancy was observed in the COVID-19 cohort reported by Karami et al. [36] (ratio 46.2), whereas the greatest absolute difference was observed in the RSV cohort reported by Wongsurakiat et al. [7] (68.6 percentage points). These findings quantify the gap between antibiotic prescribing and microbiological confirmation of bacterial co-infection across all three major viral respiratory infections and provide direct patient-level evidence of a substantial prescribing-to-confirmation gap among hospitalized adults, with particular relevance for antimicrobial stewardship programs.

However, these findings should be interpreted in the context of the inherent limitations of microbiological confirmation in clinical practice. Standard bacterial cultures have limited sensitivity particularly in patients who have received prior antibiotic therapy or who harbor fastidious or atypical organisms and may systematically underestimate the true prevalence of bacterial co-infection. In addition, clinical decision-making in hospitalized patients with severe viral respiratory disease frequently occurs under diagnostic uncertainty, where the consequences of untreated bacterial co-infection may be life-threatening. Therefore, the gap between prescribing and confirmation reflects both potential antibiotic overuse and the inherent diagnostic challenges of infectious disease practice and should not be interpreted as evidence that all empirical antibiotic prescriptions in this context are unjustified.

### 2.7. Antimicrobial Resistance Patterns

Eight studies provided direct virus-specific evidence on antimicrobial resistance (AMR) among adults hospitalized with COVID-19 [13,35,45,46,47,48,49,50]. No virus-specific AMR data were available for influenza A/B or RSV in the included evidence base. Two additional studies provided contextual AMR information from mixed viral respiratory cohorts, but their findings could not be attributed separately to influenza A/B or RSV and were therefore not used to draw virus-specific AMR conclusions [7,8]. Because AMR reporting was heterogeneous with regard to bacterial species, resistance mechanisms, timing of infection, clinical setting, and case definitions, no quantitative pooling was performed; the findings are presented as a descriptive synthesis focused on COVID-19. The eight COVID-19 studies are summarized in Table 5.

To improve consistency across studies, the infection-setting terminology used in Table 5 was standardized using epidemiological categories based on timing of infection and clinical setting. Infections reported at admission or within the first 48 h of hospitalization were classified as community-onset co-infections. Infections developing after 48 h of hospitalization were classified as hospital-acquired secondary infections. Infections specifically reported as occurring during ICU stay were classified as ICU-acquired secondary infections, whereas infections described by the original studies as healthcare-associated were classified as healthcare-associated infections. When the original study did not clearly distinguish timing or acquisition setting, the infection setting was categorized as mixed/unclear timing. In addition, resistance findings were harmonized according to standardized AMR phenotypes or mechanisms, including ESBL-producing Enterobacterales, carbapenemase-producing or carbapenem-resistant Enterobacterales, carbapenem-resistant non-fermenting Gram-negative bacilli, MDR/XDR Gram-negative organisms, and VRE when reported by the original studies. MRSA was retained only when explicitly reported but was not interpreted as a predominant AMR pattern.

Among patients hospitalized with COVID-19, eight studies provided direct evidence on AMR across diverse geographical settings, including Romania [13,47], Spain [35], Saudi Arabia [45], Brazil [46], Serbia [48], Poland [49], and Italy [50]. After standardization of infection-setting terminology, AMR findings were concentrated predominantly in hospital-acquired secondary infections, ICU-acquired secondary infections, and healthcare-associated infections. One study was categorized as mixed/unclear timing because bacterial infections were reported in ICU and non-ICU patients without a clear distinction between community-onset and hospital-acquired acquisition. Healthcare-associated MDR organisms predominated across the included COVID-19 studies, with the AMR profile being driven mainly by carbapenem-resistant Gram-negative organisms, particularly carbapenem-resistant Acinetobacter baumannii (CRAB), carbapenem-resistant Enterobacterales (CRE), carbapenemase-producing Klebsiella pneumoniae, and MDR/XDR Acinetobacter spp. MRSA was not consistently represented across the primary virus-specific AMR studies and was therefore not considered a predominant AMR pattern in this synthesis.

When AMR findings were interpreted according to clinical setting, resistant pathogens were reported predominantly in ICU/critically ill cohorts, mixed ward/ICU cohorts, or healthcare-associated infection settings. ICU/critically ill cohorts contributed particularly important evidence on carbapenem-resistant and MDR/XDR Gram-negative organisms, especially in secondary infections occurring during ICU stay. In contrast, broader hospitalized cohorts generally did not provide AMR data stratified separately for ICU and non-ICU patients. Therefore, the AMR synthesis should be interpreted as being strongly influenced by healthcare-associated and ICU-enriched evidence, rather than as a direct estimate of AMR prevalence across all adults hospitalized with viral respiratory infections.

Mateescu et al. identified MDR organisms in 46 of 112 (41.1%) secondary infections, with 82.1% of infections classified as hospital-acquired, supporting the interpretation that, in this cohort, AMR was mainly related to hospital-acquired secondary infection rather than community-onset co-infection [13]. García-Vidal et al. similarly reported MDR Gram-negative bacteria, including extended-spectrum beta-lactamase (ESBL)-producing Klebsiella pneumoniae and Pseudomonas aeruginosa, only among patients with hospital-acquired superinfection and not at admission [35]. Bazaid et al. documented extensively drug-resistant Acinetobacter baumannii and Klebsiella pneumoniae among ICU patients, with colistin being the only therapeutic option [45]. Da Costa et al. reported MDR in 96% of Acinetobacter baumannii isolates and 57% of Klebsiella pneumoniae isolates in a Brazilian ICU cohort, highlighting the severity of carbapenem-resistant Gram-negative infections in this population [46]. Dumitru et al. described nine critically ill patients with COVID-19 who developed invasive infections with carbapenemase-producing Klebsiella pneumoniae carrying KPC and/or OXA-48 enzymes, five of whom died [47]. Gajic et al. reported carbapenem resistance rates of 71.3% among Enterobacterales, 93.8% among Acinetobacter baumannii, and 69.1% among Pseudomonas aeruginosa in a Serbian multicentre ICU cohort, with most infections being hospital-acquired [48]. Pałka et al. found that extensively drug-resistant (XDR) organisms accounted for 22.6% of ICU isolates and 14.8% of non-ICU isolates, with XDR Acinetobacter spp. being the most frequent cause of healthcare-associated pneumonia [49]. Karruli et al. reported that 50% of critically ill patients with COVID-19 developed an MDR infection during their ICU stay, with a median time to MDR infection of 8 days after ICU admission [50].

Taken together, the primary virus-specific AMR evidence from COVID-19 studies indicated a predominance of carbapenem-resistant Gram-negative bacteria, particularly CRAB, CRE, carbapenemase-producing Klebsiella pneumoniae, and MDR/XDR Acinetobacter spp. These findings were concentrated mainly in secondary, ICU-associated, and healthcare-associated infections and should not be generalized to influenza A/B or RSV, for which virus-specific AMR data were not available in the included evidence base.

For influenza A/B and RSV, virus-specific AMR data were not available from the included studies. Wongsurakiat et al. reported MDR and XDR organisms in a mixed RSV and influenza cohort, with resistant bacteria being more frequent in superinfection than in co-infection present at admission; however, AMR findings could not be separated according to viral pathogen and were therefore considered contextual evidence only [7]. Similarly, Liu et al. identified MRSA as the predominant organism among laboratory-confirmed viral–bacterial co-infections in a mixed influenza and RSV cohort, but virus-specific attribution was not possible [8]. These contextual mixed-cohort findings were not used to infer AMR patterns for influenza A/B or RSV. The absence of direct virus-specific AMR data for influenza A/B and RSV represents a significant evidence gap that warrants dedicated prospective studies.

### 2.8. Clinical Outcomes

Clinical outcome data were obtained from 14 included studies, yielding virus- and exposure-specific comparisons among adults hospitalized with COVID-19, influenza A or B, or RSV infection [7,15,20,22,23,24,26,28,31,35,39,46,49]. Owing to heterogeneity in study design, exposure definitions, comparator groups, and outcome ascertainment methods, quantitative pooling could not be performed; therefore, the findings are presented as a descriptive narrative synthesis, organized according to exposure category and viral infection group, including Mateescu et al. [13] and Fu et al. [44]. A structured summary is provided in Table 6.

Three studies examining patients with COVID-19 reported that empirical antibiotic exposure without a confirmed bacterial indication was independently associated with higher rates of secondary infection, ICU admission, mechanical ventilation, and in-hospital mortality [15,20,22]. Six additional studies showed that confirmed bacterial co-infection and secondary infection were associated with substantially higher mortality, increased ICU admissions, and prolonged hospitalization, with MDR organisms frequently involved in the most severe cases [13,23,24,32,36,47,50].

For influenza, the evidence was inconsistent: two studies found no significant adjusted association between bacterial co-infection and 30-day mortality [40,41], whereas Karlsen et al. reported increased 14-day and 90-day mortality among patients with influenza A and confirmed co-infection, and found that early antibiotic treatment was associated with prolonged hospitalization and higher mortality, was not associated with improved survival in the reported observational analysis [26].

In RSV, bacterial co-infection was independently associated with a severe composite outcome in one study (aOR 1.91; 95% CI 1.36–2.69) [28] and with higher in-hospital mortality in two other cohorts [7,44]. Early antibiotic use among patients with RSV was similarly associated with prolonged hospitalization, was not associated with improved survival in this observational cohort [26].

## 3. Discussion

### 3.1. Main Findings

This systematic review and meta-analysis of 39 studies, including 839,531 hospitalized adults, highlights three consistent findings across COVID-19, influenza A/B, and RSV. First, empirical antibiotic use was highly prevalent in all three groups of acute viral infections, with pooled estimates exceeding 57% in all subgroups. Second, confirmed bacterial co-infection was significantly less frequent than antibiotic prescribing across all groups, with pooled prevalence estimates of 5.31% for COVID-19, 18.66% for influenza A/B, and 24.36% for RSV. Third, the resulting gap between antibiotic prescribing and microbiological confirmation of bacterial co-infection was large, consistent, and directly quantifiable: within-cohort prescribing-to-infection ratios ranged from 3.0 to 46.2 across seven cohorts. These findings, together with evidence that confirmed bacterial complications are associated with adverse clinical outcomes, support the need for careful reassessment and early re-evaluation of empirical antibiotic therapy in adults hospitalized with viral respiratory infections, particularly when microbiological or clinical evidence of bacterial infection is lacking. However, because the available evidence regarding empirical antibiotic exposure is predominantly observational, heterogeneous, and susceptible to confounding by indication and diagnostic misclassification, these findings should not be interpreted as proving an absence of benefit from empirical antibiotics in all hospitalized patients with viral respiratory infections.

### 3.2. Interpretation and Context

The pooled prevalence of antibiotic use in COVID-19 (62.56%) was lower than the estimate reported by Langford et al. in 2020 (71.9%) [3], and broadly comparable to the estimate reported by Alshaikh et al. in 2022 (approximately 61.77%) [5]. This pattern may reflect the gradual evolution of prescribing practices and the progressive implementation of antimicrobial stewardship interventions during the later phases of the pandemic. In contrast, the pooled prevalence of confirmed bacterial co-infection in COVID-19 (5.31%) was consistent with, or slightly higher than, previous estimates of 3.5% and 5.62%, respectively [3,5], supporting the interpretation that the gap between antibiotic prescribing and microbiological confirmation in COVID-19 is a recurrent finding across the literature rather than a study-specific artifact.

For influenza A/B, the pooled prevalence of confirmed bacterial co-infection (18.66%) exceeded the approximately 11.2% reported by Arranz-Herrero et al. [6], a difference that may be attributable to heterogeneity in co-infection definitions across studies, variability in the timing of microbiological sampling, and the inclusion of both community-acquired and hospital-acquired infections in several contributing cohorts.

For RSV, this synthesis provides the first systematic pooled estimates of both antibiotic use (76.03%) and confirmed bacterial co-infection (24.36%) in hospitalized adult populations. One of the important findings of this review is the particularly high antibiotic exposure observed in RSV cohorts, which exceeded that observed in both COVID-19 and influenza A/B in the pooled analysis. Several factors may explain this pattern. RSV in adults is increasingly recognized as a clinically significant cause of hospitalization and severe lower respiratory tract disease, particularly among older adults and medically vulnerable patients with chronic cardiopulmonary disease [1,2,7,28,29,30]. In these populations, RSV infection may present with severe dyspnea, radiological abnormalities, exacerbation of chronic airway disease, and systemic inflammatory features that overlap clinically with bacterial pneumonia, thereby increasing diagnostic uncertainty and the likelihood of empirical antibiotic initiation.

Compared with COVID-19, for which bacterial co-infection rates became better characterized during the pandemic and antimicrobial stewardship recommendations progressively evolved, RSV in adults has historically received less attention in diagnostic and stewardship algorithms. Clinicians may therefore be more likely to prescribe antibiotics empirically in hospitalized RSV patients, particularly when rapid viral diagnosis is delayed, microbiological sampling is incomplete, or bacterial co-infection cannot be confidently excluded at admission. The relatively high pooled prevalence of confirmed bacterial co-infection in RSV compared with COVID-19 may also contribute to this prescribing behavior, although antibiotic exposure still substantially exceeded microbiological confirmation in the available within-cohort data [7,28,29,30,40,44].

These findings suggest that RSV should not be viewed only as a pediatric pathogen or as a minor adult viral infection in antimicrobial stewardship programs. Instead, hospitalized adults with RSV represent a distinct stewardship population in whom early viral diagnosis, adequate microbiological sampling, structured reassessment at 48–72 h, and timely antibiotic de-escalation when bacterial infection is not confirmed may be particularly important. However, because the RSV evidence base remains limited and heterogeneous, these implications should be interpreted cautiously and require confirmation in prospective studies specifically designed to evaluate antibiotic decision-making, bacterial co-infection, and clinical outcomes in adults hospitalized with RSV.

The very high heterogeneity observed in the meta-analyses, particularly for antibiotic use and confirmed bacterial co-infection, represents an important methodological limitation and should be considered when interpreting all pooled estimates. This heterogeneity likely reflects differences in study period, pandemic phase, country and healthcare system, patient selection, ward versus ICU case mix, illness severity, microbiological testing intensity, definitions of co-infection or secondary infection, and local antibiotic prescribing practices. Consequently, the pooled estimates should not be interpreted as precise universal prevalence values or as direct comparative effects between COVID-19, influenza A/B, and RSV. Rather, they should be understood as descriptive summary estimates that identify broad patterns, especially the consistent finding that antibiotic prescribing exceeded microbiological confirmation across the included viral groups.

A further source of heterogeneity was the inconsistent use of the terms bacterial co-infection, secondary infection, and superinfection across the included studies. Conceptually, bacterial co-infection refers to microbiologically confirmed bacterial infection present at admission or detected early during hospitalization, generally within the first 48 h when timing was specified. By contrast, secondary infection and superinfection generally refer to later-onset infections developing after the initial viral illness, often in hospital-acquired or ICU-associated settings. However, these terms were not applied uniformly across studies, and the timing of microbiological sampling, diagnostic intensity, and criteria for confirmation differed substantially. For this reason, admission or early co-infection estimates were pooled only when extractable according to the original study definitions, whereas secondary infections and superinfections were primarily synthesized narratively when reported separately. This distinction is important because early co-infection informs empirical antibiotic decisions at presentation, while secondary infection and superinfection are more closely related to disease severity, duration of hospitalization, ICU exposure, invasive procedures, and nosocomial transmission.

Within-cohort analyses provide the most direct and clinically interpretable evidence of the prescribing-to-confirmation gap. Across seven cohorts from five studies, prescribing-to-infection ratios ranged from 3.0 to 46.2, indicating that, in the most extreme case, antibiotics were prescribed to nearly 46 patients for every one patient with a microbiologically confirmed bacterial infection. The greatest relative discrepancy was observed in the COVID-19 cohort reported by Karami et al., in which confirmed bacterial co-infection was documented in only 1.3% of patients despite an antibiotic prescribing rate of 60.1% [36]. The greatest absolute difference was observed in the RSV cohort reported by Wongsurakiat et al., in which the difference between antibiotic use and confirmed co-infection reached 68.6 percentage points [7].

It is important to recognize that the prescribing-to-confirmation gap quantified in this analysis reflects not only potential antibiotic overuse but also the well-recognized limitations of microbiological diagnostics in the hospital setting. Blood cultures have a sensitivity of approximately 10–20% in community-acquired pneumonia, and sputum cultures are frequently non-diagnostic due to contamination, inadequate sample quality, or prior antibiotic exposure. Atypical bacterial pathogens—including *Legionella pneumophila*, *Mycoplasma pneumoniae*, and *Chlamydophila pneumoniae*—are systematically underdetected by standard culture-based methods and require molecular or serological testing that is not universally applied across settings. Prior antibiotic exposure, which is common among hospitalized patients, further reduces culture yield and may sterilize specimens that would otherwise test positive. In this context, reported rates of confirmed bacterial co-infection in the included studies likely represent minimum estimates of true bacterial involvement, and the prescribing-to-infection ratios presented in this analysis should be interpreted accordingly. Clinical judgment in hospitalized patients with severe viral respiratory disease must also consider the risk of withholding antibiotics in the presence of clinically suspected but unconfirmed bacterial co-infection, particularly in critically ill patients in whom bacterial complications contribute substantially to attributable mortality. These considerations do not diminish the importance of effective antimicrobial stewardship in this context, but they emphasize that the goal of stewardship should be optimization of diagnostic accuracy—through rapid multiplex diagnostics, systematic microbiological sampling, and protocolized de-escalation rather than universal delay of empirical antibiotic therapy.

Antimicrobial resistance data were available exclusively from COVID-19 studies and should therefore be interpreted as COVID-19-specific evidence rather than as a pattern applicable to all hospitalized adults with viral respiratory infections. Within the COVID-19 AMR evidence base, the available studies revealed a consistent pattern of healthcare-associated multidrug-resistant Gram-negative organisms across diverse geographic settings. Carbapenem-resistant *Acinetobacter baumannii* (CRAB), carbapenem-resistant Enterobacterales (CRE), carbapenemase-producing *Klebsiella pneumoniae*, and other MDR/XDR Gram-negative organisms were the predominant resistant pathogens identified in secondary and hospital-acquired infections [13,35,45,46,47,48,49,50]. MRSA was not consistently represented across the primary virus-specific AMR studies and was therefore interpreted as a less prominent finding in this synthesis. These findings should not be generalized to adults hospitalized with influenza A/B or RSV, for whom direct virus-specific AMR data were not available in the included evidence base. This represents a major evidence gap and highlights the need for dedicated virus-specific AMR surveillance studies in adults hospitalized with influenza A/B and RSV.

An important implication of the available AMR evidence is the strong influence of ICU and healthcare-associated infection settings. ICU patients differ substantially from general hospitalized patients in terms of baseline illness severity, exposure to invasive devices, duration of hospitalization, intensity and duration of antimicrobial therapy, and risk of nosocomial transmission of multidrug-resistant organisms. Consequently, the predominance of carbapenem-resistant Gram-negative organisms observed in the COVID-19 AMR synthesis should not be interpreted as reflecting the microbiology of all adults hospitalized with COVID-19, and should not be extrapolated to influenza A/B or RSV. Rather, it most likely reflects the burden of secondary and healthcare-associated infections in critically ill or highly exposed hospitalized patients with COVID-19. This distinction is clinically important because empirical antibiotic decisions at admission in general ward patients should not be guided solely by ICU-derived COVID-19 AMR patterns, whereas ICU patients with late secondary deterioration require a different diagnostic and stewardship approach, including rapid microbiological testing and local resistance surveillance.

Regarding clinical outcomes, confirmed bacterial co-infection and secondary infection were consistently associated with higher in-hospital mortality, increased ICU admission rates, and prolonged hospitalization across the three viral infection groups. Secondary bacterial infections, particularly those acquired in hospital and caused by multidrug-resistant organisms, were associated with the most severe outcomes, including prolonged ICU stays and high mortality rates [31,35,46,50]. By contrast, studies evaluating empirical antibiotic exposure in the absence of microbiologically confirmed bacterial infection did not provide consistent evidence of improved survival, particularly in COVID-19 cohorts, and some COVID-19 studies reported associations with secondary infection or in-hospital mortality [15,20,22]. These findings should be interpreted cautiously because antibiotic exposure was not randomized and was likely influenced by illness severity, clinical suspicion of bacterial infection, microbiological testing practices, and local prescribing protocols. Therefore, the available evidence supports structured reassessment, microbiological sampling, and timely de-escalation when bacterial infection is not confirmed, rather than a broad conclusion that empirical antibiotics are uniformly ineffective or harmful in all hospitalized patients with viral respiratory infections.

### 3.3. Implications for Antimicrobial Stewardship

The virus-specific variation in the rates of confirmed bacterial co-infection identified in this work has direct and actionable implications for antimicrobial stewardship. The significantly lower burden of confirmed bacterial co-infection in COVID-19 compared with influenza A/B and RSV suggests that the threshold for discontinuing empirical antibiotics, or for avoiding their initiation at presentation when clinical features are consistent with a primary viral syndrome, should differ according to the identified pathogen. In COVID-19, in which confirmed bacterial co-infection was present in fewer than 6% of hospitalized patients, the available evidence supports a default conservative strategy of early antibiotic de-escalation guided by microbiological results. In influenza A/B and RSV, in which bacterial complications occurred in approximately one in five and one in four patients, respectively, a more cautious initial approach may be justified, with empirical therapy targeted toward the most likely pathogens and promptly reassessed once culture results become available.

Rapid multiplex respiratory diagnostic tests, combined with systematic microbiological sampling at admission, including cultures, represent the most effective tools for reducing the gap between prescribing and confirmation identified in these analyses. The presence of a confirmed viral diagnosis at presentation should be actively integrated into antibiotic decision-making algorithms, and the absence of a positive bacterial culture or a validated biomarker threshold should prompt early reassessment of empirical therapy. Adults with pre-existing chronic airway diseases, including chronic obstructive pulmonary disease (COPD) and asthma, require particular attention in this context: they constitute a disproportionately large and vulnerable subgroup across all three viral infections, have a higher likelihood of receiving empirical antibiotics at presentation, and are at greater risk of bacterial complications and associated adverse outcomes [11,12]. Virus-specific and comorbidity-adapted stewardship protocols targeting this subgroup require dedicated prospective evaluation.

### 3.4. Strengths and Limitations

To our knowledge, this is the first systematic review and meta-analysis to simultaneously synthesize antibiotic use, confirmed bacterial co-infection, antimicrobial resistance patterns, and clinical outcomes across all three major viral respiratory infections within a unified analytical framework. This approach allows direct descriptive comparisons between pathogens, which were not possible in previous reviews focused on a single virus, and provides a more complete picture of the gap between prescribing and confirmation in the context of viral respiratory disease.

Several limitations should be mentioned. Between-study heterogeneity was considerable across all subgroups (I^2^ = 67–99%), reflecting real variation in study design, patient populations, pandemic periods, clinical settings, illness severity, operational definitions of antibiotic use, bacterial co-infection, secondary infection, and superinfection, timing of infection assessment, microbiological confirmation criteria, microbiological testing practices, and local prescribing patterns. Therefore, pooled estimates should be interpreted with caution, as descriptive summary measures rather than precise or universally generalizable prevalence values. The evidence base was markedly asymmetrical, with 31 studies available for COVID-19 compared with 10 for influenza A/B and 8 for RSV, which may have reduced the statistical power and precision of estimates in the non-COVID-19 subgroups. In addition, this review relied entirely on aggregate study-level data rather than individual patient-level data. This limited our ability to perform adjusted patient-level analyses, evaluate effect modification by age, comorbidities, illness severity, ICU admission, timing of infection, or prior antibiotic exposure, and distinguish whether observed differences reflected true biological variation or differences in case mix, clinical setting, and diagnostic practices across studies.

Another methodological limitation is that the review was registered retrospectively on OSF, after initiation of the review process. Although no substantive deviations from the registered protocol were made after registration, retrospective registration limits the ability to demonstrate that all methodological decisions were prespecified before screening, extraction, and synthesis. All included studies were observational, and the reported associations between antibiotic exposure or bacterial co-infection and clinical outcomes are subject to confounding by indication, as antibiotic treatment was frequently initiated in patients with greater disease severity or higher clinical suspicion of bacterial infection, making causal inference impossible without randomized evidence. This limitation is particularly important because no included study was judged to have a low risk of bias, and 13 studies had major methodological concerns, which reduces confidence in the precision and generalizability of the pooled and narrative findings. Accordingly, the absence of consistent evidence of improved survival among patients receiving empirical antibiotics without microbiological confirmation should be considered hypothesis-generating rather than causal evidence, because illness severity, diagnostic uncertainty, selective microbiological testing, and imperfect sensitivity of culture-based diagnostic methods may have influenced both antibiotic prescribing and outcome assessment.

Rates of confirmed bacterial co-infection across all three viral groups probably underestimate the true prevalence, given the inconsistent and often clinician-directed microbiological testing. In addition, the terms bacterial co-infection, secondary infection, and superinfection were not used uniformly across the included studies, and timing of infection onset was not always clearly reported. This limited the direct comparability of bacterial infection outcomes across studies and may have contributed to heterogeneity in the pooled estimates. Furthermore, ICU and healthcare-associated infection settings were over-represented in the available AMR evidence, limiting the generalizability of resistance findings to broader non-ICU hospitalized populations. Moreover, AMR conclusions are primarily COVID-19-specific because no direct virus-specific AMR data were available for influenza A/B or RSV in the included evidence base. Therefore, the predominance of carbapenem-resistant Gram-negative organisms should be interpreted primarily in the context of critically ill patients with COVID-19, secondary infections, and healthcare-associated infections, rather than as a direct reflection of AMR patterns among all adults hospitalized with viral respiratory infections. Finally, a formal GRADE certainty-of-evidence assessment was not performed, because the review combined prevalence meta-analyses with descriptive and narrative syntheses of AMR patterns and clinical outcomes across heterogeneous observational studies. Confidence in the evidence was instead considered through risk-of-bias assessment, sensitivity analyses, heterogeneity assessment, and cautious interpretation of the findings.

### 3.5. Future Research

Priority areas for future research include prospective studies using standardized microbiological protocols for influenza and RSV; dedicated AMR surveillance studies in non-SARS-CoV-2 viral respiratory infections; randomized or quasi-experimental evaluations of the impact of rapid diagnostic algorithms on antibiotic prescribing; and prospective cohort studies examining the role of chronic airway diseases, particularly COPD and asthma, as modifiers of the risk of bacterial co-infection, antibiotic prescribing, and the emergence of AMR among adults hospitalized with viral respiratory infections.

## 4. Materials and Methods

### 4.1. Study Design

This systematic review and meta-analysis was conducted and reported in accordance with the Preferred Reporting Items for Systematic Reviews and Meta-Analyses (PRISMA) 2020 guidelines. The review was retrospectively registered on the Open Science Framework (OSF; available at: https://osf.io/dga3y accessed on 4 June 2026). Because registration was completed retrospectively, after initiation of the review process, this is explicitly acknowledged as a methodological limitation. No substantive deviations from the registered protocol were made after OSF registration. A completed PRISMA 2020 checklist is provided as Supplementary File S1.

### 4.2. Search Strategy

A systematic literature search was conducted in four electronic databases—PubMed/MEDLINE, Embase, the Cochrane Central Register of Controlled Trials (CENTRAL), and Web of Science—in March 2026. The search strategies were developed using a combination of Medical Subject Headings (MeSH) terms and free-text keywords organized around three conceptual domains: (1) viral respiratory infection (COVID-19, SARS-CoV-2, influenza, influenza A, influenza B, respiratory syncytial virus, RSV); (2) bacterial co-infection, secondary bacterial infection, antibiotic use, antimicrobial prescribing, or antimicrobial resistance; and (3) hospitalized adults, inpatients, or hospital admission. The complete search strategy for all four databases is provided in Supplementary File S2. The reference lists of all included studies were manually screened to identify additional eligible records. Gray literature databases, preprint servers, dissertations, theses, institutional reports, and unpublished studies were not searched, because the review was restricted to full-text peer-reviewed primary studies. No language restrictions or date limitations were applied during the database searches or study selection process.

### 4.3. Eligibility Criteria

Studies were included if they met all of the following criteria: (i) they enrolled adult patients (≥18 years) hospitalized with laboratory-confirmed COVID-19, influenza A or B, or respiratory syncytial virus (RSV) infection, confirmed by reverse transcription polymerase chain reaction (RT-PCR) or validated antigen testing; (ii) theygrayorted at least one of the following outcomes: prevalence of antibiotic use or prescribing, prevalence of confirmed bacterial co-infection or secondary bacterial infection, antimicrobial resistance patterns, or clinical outcomes (mortality, ICU admission, mechanical ventilation, or length of hospital stay) associated with antibiotic exposure or bacterial complications; and (iii) they employed an observational study design (prospective or retrospective cohort, analytical cross-sectional study, or case series) or were randomized controlled clinical trials. Studies enrolling exclusively pediatric populations were excluded; mipediatricpaediatric cohorts were included only if adult-specific data could be extracted separately or if adults represented ≥80% of participants. Systematic reviews, narrative reviews, case reports, editorials, commentaries, letters, and conference abstracts or proceedings without a corresponding full-text peer-reviewed article were excluded. Unpublished or non-peer-reviewed literature, including preprints, dissertations, theses, institutional reports, and other grey literature sources, was not eligible for inclusion. Only full-text peer-reviewed primary studies reporting extractable data for at least one prespecified outcome were included. Non-English studies were eligible for inclusion, provided that they were full-text peer-reviewed primary studies and contained sufficient extractable data for at least one prespecified outcome; however, all studies ultimately included in the final synthesis were published in English.

### 4.4. Study Selection and Data Extraction

All records retrieved from the database searches were exported and compiled in Microsoft Excel (Microsoft Corporation, Redmond, WA, USA), where deduplication was performed manually. Titles and abstracts were screened by the primary reviewer against the predefined eligibility criteria; a random sample of records was independently verified by a second reviewer to ensure consistency, and disagreements were resolved through discussion. Full-text assessment was subsequently performed for all records not excluded at the title and abstract screening stage, following the same process. Data were extracted by the primary reviewer using a standardized extraction system that captured study characteristics—first author, year, country, design, study period, and clinical setting patient demographic data, age, sex, and comorbidities viral pathogen and confirmation method, definition and prevalence of antibiotic use, definition, timing, and prevalence of bacterial co-infection, antimicrobial resistance patterns and organisms, and clinical outcome data, including comparator groups and effect estimates.

For the purpose of data extraction and synthesis, bacterial infection outcomes were categorized according to timing and microbiological confirmation whenever this information was available. Confirmed bacterial co-infection was defined as a microbiologically confirmed bacterial infection reported at admission or within the first 48 h of hospitalization or viral diagnosis. Secondary bacterial infection was defined as a microbiologically confirmed bacterial infection developing after 48 h of hospitalization or later during the clinical course. Bacterial superinfection was defined as a later-onset or hospital-acquired bacterial infection occurring after the initial viral illness, as reported by the original study authors; when timing was consistent with onset after 48 h, superinfection was grouped descriptively with secondary infection. When original studies used overlapping terminology or did not clearly distinguish timing, the original definition was retained and the outcome was categorized as mixed or unclear timing. Clinically suspected bacterial infections without microbiological confirmation were not included in the quantitative synthesis of confirmed bacterial co-infection, but were considered in the narrative synthesis when reported as part of clinical outcome analyses.

Extracted data were independently verified by a second reviewer, and disagreements were resolved through consensus. For studies contributing data on more than one eligible viral infection, data for each pathogen were extracted and analyzed as independent cohorts.

### 4.5. Quality Assessment

The methodological quality of each included study was independently assessed by two reviewers using the Joanna Briggs Institute (JBI) critical appraisal tools appropriate to each study design: the JBI checklist for cohort studies was applied to cohort designs; the JBI checklist for analytical cross-sectional studies was applied to cross-sectional studies and administrative database designs; the JBI checklist for case series was applied to case series; and the JBI checklist for studies reporting prevalence data was applied to laboratory-based prevalence studies.

Each study was classified as having low, moderate, or high methodological concern based on the proportion and clinical significance of unmet criteria. Disagreements were resolved through consensus. Detailed study-level assessments are presented in Supplementary Table S2.

### 4.6. Statistical Analysis

Quantitative synthesis was performed for two primary outcomes: reported antibiotic use (19 studies, 21 virus-specific cohorts) and confirmed bacterial co-infection (17 studies, 22 virus-specific cohorts).

Pooled prevalence estimates were calculated using random-effects meta-analysis. Given the bounded nature of proportional data, the Freeman–Tukey double-arcsine transformation was applied before pooling to stabilize variance. Back-transformed estimates are presented as pooled proportions with 95% confidence intervals. Between-study heterogeneity was quantified using the I^2^ statistic and Cochran’s Q test. I^2^ values were interpreted as follows: low, below 25%; moderate, between 25% and 50%; substantial, between 50% and 75%; and considerable, above 75%. Prespecified subgroup analyses were conducted according to viral pathogen COVID-19, influenza A/B, and RSV as the primary unit of reporting, given the anticipated degree of overall heterogeneity.

Prespecified sensitivity analyses were performed by restricting each subgroup to studies assessed as having moderate methodological concerns, in order to evaluate the robustness of the primary estimates after excluding studies with high methodological concerns. Publication bias was assessed for subgroups contributing ten or more cohorts using Egger’s test and Begg’s test. No formal assessment was performed for subgroups with fewer than ten contributing studies.

For antimicrobial resistance patterns and clinical outcomes, no quantitative pooling was performed because of heterogeneity in outcome definitions, comparator groups, and reporting formats. Findings are presented as descriptive narrative syntheses. All statistical analyses were performed using MedCalc Statistical Software, version 23.5.5 (MedCalc Software Ltd., Ostend, Belgium).

### 4.7. Certainty of Evidence Assessment

A formal certainty-of-evidence assessment using GRADE was not performed. This decision was made because the review primarily synthesized prevalence estimates and descriptive evidence on antibiotic use, confirmed bacterial co-infection, antimicrobial resistance patterns, and clinical outcomes rather than evaluating a single comparative intervention effect. In addition, the included studies were observational and highly heterogeneous in terms of study design, clinical setting, outcome definitions, microbiological testing practices, and reporting structure. Several outcomes, particularly antimicrobial resistance patterns and clinical outcomes, were summarized narratively rather than quantitatively. Therefore, applying a formal GRADE framework across all outcomes was considered methodologically inappropriate and potentially misleading. Instead, confidence in the evidence was addressed through study-level risk-of-bias assessment using Joanna Briggs Institute tools, prespecified sensitivity analyses restricted to studies with moderate methodological concerns, assessment of statistical heterogeneity, and cautious interpretation of findings in the Discussion and Limitations sections.

## 5. Conclusions

Although pooled estimates should be interpreted cautiously because of very high between-study heterogeneity, this systematic review and meta-analysis shows that antibiotic use is highly prevalent among adults hospitalized with COVID-19, influenza A/B, or RSV and substantially exceeds the rates of confirmed bacterial co-infection across all three viral infection groups. The large and consistent prescribing-to-confirmation gap, with prescribing-to-infection ratios ranging from 3.0 to 46.2 in within-cohort analyses, suggests substantial potential empirical antibiotic overuse, although this finding should be interpreted in the context of diagnostic uncertainty, heterogeneous microbiological testing, the observational nature of the available evidence, and the absence of studies at low risk of bias. When bacterial complications occur, they are associated with worse clinical outcomes, including higher mortality, ICU admission, and prolonged hospitalization. In patients with COVID-19, available AMR data indicate a predominance of carbapenem-resistant Gram-negative organisms, particularly in secondary, ICU-associated, and healthcare-associated infections. These AMR findings should be interpreted as COVID-19-specific and should not be extrapolated to adults hospitalized with influenza A/B or RSV, for whom comparable virus-specific AMR data remain lacking. The particularly high antibiotic exposure observed in RSV cohorts suggests that hospitalized adults with RSV should be specifically addressed in antimicrobial stewardship strategies, especially older and medically vulnerable patients in whom viral disease may closely mimic bacterial pneumonia or chronic cardiopulmonary decompensation. Overall, these findings support virus-specific antimicrobial stewardship strategies in adult inpatient settings, with rapid viral and bacterial diagnosis, systematic microbiological sampling, structured reassessment, and timely de-escalation when bacterial infection is not confirmed. Because no included study was judged to have a low risk of bias and several studies had major methodological concerns, these findings should be interpreted as clinically important but methodologically cautious evidence rather than as definitive causal evidence. Prospective studies using standardized diagnostic and reporting protocols are needed to address the remaining evidence gaps regarding bacterial complications, antibiotic decision-making, and resistance patterns associated with influenza A/B and RSV.

## Figures and Tables

**Table 1:** Principal characteristics of studies included in the systematic review and meta-analysis.

No.	First Author (Year) [Ref.]	Country/Region	Viral Infection(s)	N	Outcome Contribution
1	Mateescu et al. (2026) [13]	Romania	COVID-19	395	AU; BCI; PC; AMR; CO
2	Lima and Romero (2026) [14]	Brazil	COVID-19	638	AU
3	Friedrichs et al. (2025) [15]	Germany	COVID-19	1317	AU; CO
4	Elatris et al. (2025) [16]	Oman	COVID-19	176	AU; BCI; PC; CO
5	Pulia et al. (2025) [17]	United States	COVID-19; influenza; RSV; other respiratory viruses	620,630	AU
6	Blais et al. (2023) [18]	Hong Kong	COVID-19	65,810	AU
7	Sili et al. (2024) [19]	International; 28 countries	COVID-19	7830	AU; BCI
8	Pinte et al. (2022) [20]	Romania	COVID-19	553	AU; CO
9	Murillo-Zamora et al. (2022) [21]	Mexico	COVID-19	214,171	AU
10	Calderon-Parra et al. (2021) [22]	Spain	COVID-19	13,932	AU; CO
11	Vaughn et al. (2021) [23]	United States; Michigan	COVID-19	1705	AU; BCI; PC; CO
12	O’Kelly et al. (2021) [24]	Ireland	COVID-19	292	AU; BCI; CO
13	Akers et al. (2017) [25]	Switzerland	Influenza A/B	126	AU; CO
14	Karlsen et al. (2026) [26]	Denmark	RSV; influenza A/B	986	AU; BCI; CO
15	Steger et al. (2026) [27]	Sweden	Influenza A/B	174	AU; BCI
16	Haeberer et al. (2024) [28]	Spain; Valladolid	RSV; influenza; COVID-19	1364 total; 1293 hospitalized	AU; CO, BCI
17	Wongsurakiat et al. (2023) [7]	Thailand	RSV	175	AU; BCI; AMR-C; CO
18	Homen Fernandez et al. (2025) [29]	Spain; Madrid	RSV; COVID-19	200	AU; CO
19	Volling et al. (2014) [30]	Canada; Toronto	RSV	86	AU; BCI; PC; CO
20	Duan et al. (2024) [31]	China; Chengdu	COVID-19	1091	AU; BCI; PC; CO
21	Antuori et al. (2023) [32]	Spain	COVID-19	2121	AU; BCI; PC; CO
22	Moreno-García et al. (2022) [33]	Spain; Barcelona	COVID-19	1125	BCI
23	Giannella et al. (2022) [34]	Italy	COVID-19	1733	AU; BCI; PC; CO
24	Garcia-Vidal et al. (2021) [35]	Spain; Barcelona	COVID-19	989	AU; BCI; PC; AMR; CO
25	Karami et al. (2021) [36]	The Netherlands	COVID-19	925	AU; BCI; PC; CO
26	Wang et al. (2021) [37]	United Kingdom; North West London	COVID-19	1396	AU; BCI; PC; CO
27	Hughes et al. (2020) [38]	United Kingdom; London	COVID-19	836	BCI; CO
28	Schoettler et al. (2023) [39]	Germany	COVID-19; influenza virus	190; 114 COVID-19 and 76 influenza	BCI; CO
29	Hedberg et al. (2022) [40]	Sweden; Stockholm	COVID-19; influenza; RSV	2260	AU; BCI; PC; CO
30	Jorda et al. (2024) [41]	Austria, Vienna	COVID-19; influenza	1337	BCI; CO
31	Bergbrant et al. (2025) [42]	Sweden	Influenza A/B	735	CO
32	Gong et al. (2025) [43]	China; Jiangsu	Influenza A	53	BCI; CO
33	Fu et al. (2025) [44]	Taiwan	RSV	36	BCI; CO
34	Bazaid et al. (2022) [45]	Saudi Arabia; Ha’il	COVID-19	108	BCI; AMR
35	da Costa et al. (2022) [46]	Brazil	COVID-19	191	AU; BCI; AMR; CO
36	Dumitru et al. (2021) [47]	Romania	COVID-19	9	AU; BCI; AMR; CO
37	Gajic et al. (2023) [48]	Serbia	COVID-19	6478	AU; BCI; AMR; CO
38	Palka et al. (2023) [49]	Poland; Krakow	COVID-19	292	BCI; AMR
39	Karruli et al. (2021) [50]	Italy; Naples	COVID-19	32	AU; BCI; AMR; CO

Abbreviations: AU—antibiotic use; BCI—confirmed bacterial co-infection; PC—paired within-cohort comparison between antibiotic use and bacterial co-infection within the same patient population; AMR—antimicrobial resistance patterns; AMR-C—contextual antimicrobial resistance evidence from mixed viral respiratory cohorts without virus-specific attribution; these studies were not used to infer AMR patterns for influenza A/B or RSV; CO—clinical outcomes (mortality, ICU admission, mechanical ventilation, or length of hospitalization); N—number of patients; Studies contributing data for more than one eligible viral infection are listed only once; contributions to outcomes are reported separately for each viral pathogen, where applicable.

**Table 2:** Random-effects pooled prevalence of reported antibiotic use among adults hospitalized with COVID-19, influenza A/B, or respiratory syncytial virus (RSV) infection.

Viral Infection Group	Virus-Specific Cohorts, *n*	Hospitalized Adults, N	Pooled Prevalence, %	95% CI	I^2^, %	*p*-Value for Heterogeneity
Overall	21	823,874	65.18	58.26–71.79	99.96	<0.0001
COVID-19	13	821,592	62.56	53.75–70.97	99.97	<0.0001
Influenza A/B	3	934	57.48	25.76–86.09	98.7	<0.0001
RSV	5	1348	76.03	67.62–83.53	90.2	<0.0001

**Table 3:** Random-effects pooled prevalence of confirmed bacterial co-infection among adults hospitalized with COVID-19, influenza A/B, or respiratory syncytial virus (RSV) infection.

Viral Infection Group	Virus-Specific Cohorts, *n*	Hospitalized Adults, N	Pooled Prevalence, %	95% CI	I^2^, %	*p*-Value for Heterogeneity
Overall	22	16,309	11.04	7.83–14.72	97.93	<0.0001
COVID-19	12	13,471	5.31	3.43–7.56	96.54	<0.0001
Influenza A/B	6	2168	18.66	9.98–29.30	96.64	<0.0001
RSV	4	670	24.36	18.53–30.70	67.68	0.0258

Note: Confirmed bacterial co-infection refers to microbiologically confirmed bacterial infection reported at admission or within the first 48 h of hospitalization whenever timing was specified. Studies using broader, composite, or unclear definitions were retained according to the original study definition and interpreted cautiously.

**Table 4:** Within-cohort descriptive comparison of reported antibiotic use and confirmed bacterial co-infection in the same patient population.

First Author (Year) [Ref.]	Virus	Clinical Setting	N	Antibiotic Use, %	Confirmed BCI, %	Absolute Gap, pp	Ratio (AU/BCI)
COVID-19 cohorts							
Vaughn et al. (2021) [23]	COVID-19	Mixed ward/ICU	1705	56.6	3.5	53.1	16.2×
Antuori et al. (2023) [32]	COVID-19	General ward	1157	42.33	6.9	35.43	6.1x
Karami et al. (2021) [36]	COVID-19	General ward	925	60.1	1.3	58.8	46.2×
Hedberg et al. (2022) [40]	COVID-19	Mixed ward/ICU	1243	33	4	29	8.25×
Influenza A/B cohort							
Hedberg et al. (2022) [40]	Influenza A/B	Mixed ward/ICU	775	84	27	57	3.1×
RSV cohorts							
Wongsurakiat et al. (2023) [7]	RSV	General ward	175	85.7	17.1	68.6	5.0×
Hedberg et al. (2022) [40]	RSV	Mixed ward/ICU	242	88	29	59	3.0×
All 7 cohorts—range	All	—	—	33–88%	1.3–29%	29–68.6	3.0×–46.2×

Abbreviations: AU—reported antibiotic use; BCI—confirmed bacterial co-infection, generally reported at admission or within the first 48 h of hospitalization; when timing was unclear, the original study definition was retained; COVID-19—coronavirus disease 2019; pp—percentage points; ICU—intensive care unit; N—number of patients in the shared cohort; RSV—respiratory syncytial virus. Absolute gap = AU (%) − confirmed BCI (%); a positive value indicates that antibiotic prescribing exceeded the rate of confirmed bacterial co-infection. Ratio (AU/BCI) = AU (%) ÷ confirmed BCI (%); a ratio > 1 indicates that antibiotics were prescribed at a rate exceeding the rate of confirmed bacterial infection within the same cohort.

**Table 5:** Standardized descriptive synthesis of antimicrobial resistance patterns reported in COVID-19 studies.

First Author (Year) [Ref.]	Country	Clinical Setting Category	Virus	Standardized Epidemiological Infection Setting	Standardized AMR Phenotype/Mechanism and Representative Organisms	Main AMR Finding
Mateescu et al. (2026) [13]	Romania	Mixed ward/ICU or not separately stratified	COVID-19	Hospital-acquired secondary infection (>48 h after admission; predominantly hospital-acquired)	ESBL-producing Enterobacterales; carbapenemase-producing/carbapenem-resistant Enterobacterales (KPC/OXA-48-producing Klebsiella pneumoniae); carbapenem-resistant Acinetobacter baumannii (CRAB); multidrug-resistant Pseudomonas aeruginosa; vancomycin-resistant Enterococcus faecium (VRE)	MDROs were isolated in 46/112 (41.1%) secondary infections; 92/112 (82.1%) secondary infections were hospital-acquired, supporting the interpretation that, in this cohort, AMR was mainly related to hospital-acquired secondary infection rather than community-onset co-infection
García-Vidal et al. (2021) [35]	Spain	Mixed hospitalized cohort	COVID-19	Hospital-acquired secondary infection/superinfection (>48 h after admission)	ESBL-producing Enterobacterales (Escherichia coli and Klebsiella pneumoniae); multidrug-resistant Pseudomonas aeruginosa	Multidrug-resistant Gram-negative bacteria were isolated in 7 patients with hospital-acquired superinfection, supporting concentration of AMR findings in secondary infections rather than admission co-infections
Bazaid et al. (2022) [45]	Saudi Arabia	Mixed ICU/non-ICU; not separately stratified	COVID-19	Mixed/unclear timing; ICU and non-ICU bacterial infections reported without distinction between community-onset and hospital-acquired infection	Extensively drug-resistant (XDR) Gram-negative organisms, including Acinetobacter baumannii and Klebsiella pneumoniae	ICU isolates showed extensive resistance; Acinetobacter baumannii and Klebsiella pneumoniae were predominant and were reported as resistant to all tested antibiotics except colistin
da Costa et al. (2022) [46]	Brazil	ICU/critically ill	COVID-19	ICU-acquired secondary infection	Multidrug-resistant Gram-negative organisms, predominantly multidrug-resistant Acinetobacter baumannii and Klebsiella pneumoniae; carbapenem-resistant Gram-negative phenotype	Secondary infections were predominantly Gram-negative; multidrug resistance was reported in 96% of Acinetobacter baumannii and 57% of Klebsiella pneumoniae isolates
Dumitru et al. (2021) [47]	Romania	ICU/critically ill	COVID-19	ICU-acquired secondary invasive infection	Carbapenemase-producing Enterobacterales, specifically KPC- and/or OXA-48-producing Klebsiella pneumoniae	Nine severe ICU COVID-19 patients developed invasive infection with carbapenemase-producing Klebsiella pneumoniae carrying KPC and/or OXA-48; five patients died
Gajic et al. (2023) [48]	Serbia	Mixed hospitalized cohort	COVID-19	Healthcare-associated infection; predominantly hospital-acquired	Carbapenem-resistant Enterobacterales (CRE); carbapenem-resistant Acinetobacter baumannii (CRAB); carbapenem-resistant Pseudomonas aeruginosa	Most bacterial infections were hospital-acquired; carbapenem resistance was 71.3% in Enterobacterales, 93.8% in Acinetobacter baumannii and 69.1% in Pseudomonas aeruginosa
Pałka et al. (2023) [49]	Poland	Mixed ICU/non-ICU	COVID-19	Healthcare-associated infection; ICU and non-ICU settings	Extensively drug-resistant (XDR) Gram-negative organisms, predominantly XDR Acinetobacter spp./Acinetobacter baumannii; carbapenem-resistant Enterobacterales	XDR organisms represented 22.6% of ICU isolates and 14.8% of non-ICU isolates; XDR phenotypes were most frequent among Acinetobacter spp. causing pneumonia
Karruli et al. (2021) [50]	Italy	ICU/critically ill	COVID-19	ICU-acquired secondary MDR infection during ICU stay	Carbapenem-resistant Enterobacterales, including carbapenem-resistant Klebsiella pneumoniae; multidrug-resistant Acinetobacter baumannii	Fifty percent of critically ill COVID-19 patients developed an MDR infection during ICU stay after a median of 8 days; the most common MDR pathogens caused bloodstream infections and pneumonia

Note: The table includes only studies providing direct virus-specific AMR evidence from COVID-19 cohorts. Contextual AMR findings from mixed influenza/RSV cohorts were not included in this table because AMR data could not be attributed separately to individual viral pathogens.

**Table 6:** Clinical outcomes associated with antibiotic exposure, bacterial co-infection, secondary infection, or MDR infection in adults hospitalized with COVID-19, influenza, or RSV.

First Author (Year) [Ref.]	Virus	Exposure Category	Comparator	Outcomes Evaluated	Key Finding	Adjusted Analysis
COVID-19—Antibiotic Exposure and Outcomes						
Friedrichs et al. (2025) [15]	COVID-19	Antibiotic therapy at baseline for suspected pulmonary bacterial co-/superinfection	No baseline antibiotic therapy	Clinical deterioration at 14 days; in-hospital clinical course	In patients with moderate COVID-19, baseline antibiotic therapy was associated with substantially higher odds of clinical deterioration after 14 days (adjusted OR 5.00; 95% CI 2.50–10.93), without evidence of improved clinical outcomes in the reported observational analysis	Yes
Pinte et al. (2022) [20]	COVID-19	Antibiotic exposure without documented indication	No antibiotic exposure	In-hospital mortality	Among patients without documented criteria for antibiotic prescription, antibiotic exposure remained associated with higher in-hospital mortality after adjustment (OR 10.3; 95% CI 2–52)	Yes
Calderón-Parra et al. (2021) [22]	COVID-19	Systemic non-macrolide antibiotic exposure	No antibiotic exposure	Potential antibiotic-related complications excluding acute kidney injury	Antibiotic-treated patients had more potential drug-related complications than untreated patients (4.9% vs. 2.7%; OR 1.84; 95% CI 1.45–2.32)	No
COVID-19—Bacterial Co-infection/Secondary Infection and Outcomes						
Vaughn et al. (2021) [23]	COVID-19	Confirmed community-onset bacterial infection	No confirmed community-onset bacterial infection	In-hospital mortality; length of stay	Patients with confirmed community-onset bacterial infection had higher in-hospital mortality (47.5% vs. 18.0%) and longer hospital stay (median 7 vs. 5 days)	No
O’Kelly et al. (2021) [24]	COVID-19	Confirmed bacterial infection or other bacterial infectious complication	No antibiotic exposure	Length of stay; complications; ICU admission; mortality	Patients with confirmed bacterial infection or bacterial infectious complications had worse clinical outcomes than patients receiving no antibiotics or empirical antibiotics for pneumonia	No
Duan et al. (2024) [31]	COVID-19	Confirmed bacterial coinfection within 48 h	No bacterial infection	In-hospital mortality; ICU admission; mechanical ventilation; tracheal intubation; LOS	Bacterial coinfection was associated with higher mortality (17.4% vs. 9.5%), ICU admission (21.3% vs. 8.2%), mechanical ventilation (37.1% vs. 25.5%) and intubation (15.7% vs. 6.6%)	No
Duan et al. (2024) [31]	COVID-19	Secondary bacterial infection after >48 h	No bacterial infection	In-hospital mortality; ICU admission; mechanical ventilation; tracheal intubation; LOS	Secondary infection was associated with markedly higher mortality (28.2% vs. 9.5%), ICU admission (43.6% vs. 8.2%), mechanical ventilation (55.5% vs. 25.5%) and longer LOS (22.06 vs. 15.93 days)	No
García-Vidal et al. (2021) [35]	COVID-19	Hospital-acquired bacterial superinfection	No documented infection	Length of stay; mortality; clinical outcomes	Hospital-acquired bacterial superinfection was associated with significantly prolonged hospital stay and higher mortality	No
da Costa et al. (2022) [46]	COVID-19	Secondary ICU-acquired bacterial infection	No secondary infection	ICU length of stay; mechanical ventilation duration; in-hospital mortality	Secondary infection was associated with longer ICU stay (40 vs. 17 days), longer mechanical ventilation duration (24 vs. 9 days) and higher mortality; MDR Gram-negative organisms were frequent	No
Karruli et al. (2021) [50]	COVID-19	MDR infection during ICU stay	No MDR infection	ICU length of stay; ICU mortality	MDR infection was associated with longer ICU stay but not worse mortality; the reported crude association showed lower ICU mortality in the MDR group (OR 0.439; 95% CI 0.251–0.763), requiring cautious interpretation	No
Influenza A/B—Bacterial Co-infection and Outcomes						
Schoettler et al. (2023) [39]	Influenza	Bacterial co-infection or superinfection in ICU pneumonia	No bacterial co-infection or superinfection	Mortality	Bacterial co-/superinfection was not an independent predictor of mortality in critically ill influenza pneumonia	Yes
Hedberg et al. (2022) [40]	Influenza	Confirmed bacterial co-infection at admission	No bacterial co-infection	ICU admission; 30-day mortality	No statistically significant adjusted association between bacterial co-infection and 30-day mortality was demonstrated	Yes
Karlsen et al. (2026) [26]	Influenza B	Confirmed bacterial co-infection within 48 h	Tested negative for bacterial co-infection	Mortality at 14, 30 and 90 days; high-flow oxygen; mechanical ventilation; LOS	Bacterial co-infection was associated with prolonged LOS in patients with influenza B	Yes
Karlsen et al. (2026) [26]	Influenza A/B	Early antibiotic treatment initiated within 48 h	No early antibiotic treatment	Mortality; high-flow oxygen; mechanical ventilation; LOS	Early antibiotic treatment was associated with prolonged LOS, greater use of high-flow oxygen therapy and increased mortality in influenza A	Yes
RSV—Bacterial Co-infection and Outcomes						
Haeberer et al. (2024) [28]	RSV	Any documented coinfection, including bacterial, viral or fungal	No coinfection	Composite severe outcome: LOS ≥11 days; ICU admission; in-hospital death; readmission <30 days	Among hospitalized RSV patients, coinfection was independently associated with higher odds of severe outcome (adjusted OR 1.91; 95% CI 1.36–2.69)	Yes
Wongsurakiat et al. (2023) [7]	RSV	Confirmed bacterial coinfection within 48 h	No bacterial coinfection	30-day mortality; length of stay; hospital-free days	Bacterial coinfection was associated with higher 30-day mortality (16.7% vs. 5.5%), longer LOS (median 13 vs. 9 days) and fewer hospital-free days (median 16 vs. 21 days)	No
Karlsen et al. (2026) [26]	RSV	Early antibiotic treatment initiated within 48 h	No early antibiotic treatment	Mortality; high-flow oxygen; mechanical ventilation; LOS	Early antibiotic treatment was associated with prolonged LOS and was not associated with improved survival in the reported observational analysis	Yes

Note: Co-infection generally refers to bacterial infection present at admission or within 48 h of hospitalization; secondary infection refers to bacterial infection developing after 48 h or later during hospitalization; superinfection refers to later-onset or hospital-acquired infection as defined by the original studies. When timing could not be clearly distinguished, the original study terminology was retained.

## Data Availability

All data analyzed in this systematic review are derived from previously published studies included in the review. No new datasets were generated or analyzed. Further details can be obtained, upon request, from the primary author or the corresponding authors.

## Supplementary Materials

The following supporting information can be downloaded at: https://www.mdpi.com/article/10.3390/antibiotics15070654/s1, Table S1: Characteristics of included studies: full extracted data for all 39 studies, including study design, country, clinical setting, study period, patient demographics, viral confirmation method, and reported outcomes; Table S2: Risk of bias assessment of included studies using design-appropriate Joanna Briggs Institute (JBI) critical appraisal tools. Studies were classified as moderate or high/major methodological concern; no included study was judged to have a low risk of bias; Figure S1: Forest plot of the sensitivity analysis for reported antibiotic use in the COVID-19 subgroup (12 cohorts, excluding O’Kelly et al. [24]); Figure S2: Forest plot of the sensitivity analysis for confirmed bacterial co-infection in the COVID-19 subgroup (10 cohorts, excluding Moreno-García et al. [33] and Schoettler et al. [39]); File S1: PRISMA 2020 checklist, based on Page et al. [51]; File S2: Complete search strategy for all four electronic databases (PubMed/MEDLINE, Embase, Cochrane CENTRAL, and Web of Science).

## Abbreviations

aHR	adjusted hazard ratio
AMR	antimicrobial resistance
aOR	adjusted odds ratio
ARDS	acute respiratory distress syndrome
AU	antibiotic use
BALF	bronchoalveolar lavage fluid
BCI	bacterial co-infection
CAP	community-acquired pneumonia
CI	confidence interval
COPD	chronic obstructive pulmonary disease
COVID-19	coronavirus disease 2019
CRAB	carbapenem-resistant Acinetobacter baumannii
CRE	carbapenem-resistant Enterobacterales
CRKP	carbapenem-resistant Klebsiella pneumoniae
CRP	C-reactive protein
DOT	days of therapy
ESBL	extended-spectrum β-lactamase
HAI	healthcare-associated infection
ICU	intensive care unit
IMV	invasive mechanical ventilation
IQR	interquartile range
I^2^	inconsistency index (between-study heterogeneity)
JBI	Joanna Briggs Institute
KPC	Klebsiella pneumoniae carbapenemase
LOS	length of hospital stay
MDR	multidrug-resistant
MeSH	Medical Subject Headings
MRSA	methicillin-resistant Staphylococcus aureus
MV	mechanical ventilation
OR	odds ratio
OXA-48	oxacillinase-48
PCT	procalcitonin
PRISMA	Preferred Reporting Items for Systematic Reviews and Meta-Analyses
RR	risk ratio
RSV	respiratory syncytial virus
RT-PCR	reverse transcription polymerase chain reaction
SARS-CoV-2	severe acute respiratory syndrome coronavirus 2
SD	standard deviation
VRE	vancomycin-resistant Enterococcus
XDR	extensively drug-resistant
