# Supplementary material for: Antibiotic Use, Bacterial Co-Infection, and Antimicrobial Resistance in Adults Hospitalized with COVID-19, Influenza, or RSV: A Systematic Review and Meta-Analysis
Source: Antibiotics (Basel). 2026 Jun 30;15(7):654. doi: 10.3390/antibiotics15070654 (PMC13406035; doi:10.3390/antibiotics15070654)
Supplement: Supplementary file 1 [file antibiotics-15-00654-s001.zip › Supplementary file S2.pdf]

Supplementary File S1

Complete Search Strategy

Antibiotic Use, Bacterial Co-Infection, and Antimicrobial Resistance in Adults Hospitalized with COVID-19, Influenza, or RSV: A Systematic Review and Meta-Analysis

1. Search Overview

- Databases: PubMed/MEDLINE, Embase, Cochrane Central Register of Controlled Trials (CENTRAL), and Web of Science.
- Search Period: No date restrictions were applied; all records from database inception to March 2026 were eligible.
- Language: No language restrictions were applied; all studies included in the final synthesis were published in English.
- Design Filters: Eligible study designs included prospective and retrospective cohort studies, analytical cross-sectional studies, case series, and randomized controlled clinical trials. Systematic reviews, case reports, editorials, commentaries, and conference abstracts without full-text data were excluded.

2. Database-Specific Search Strings

Database 1: PubMed/MEDLINE

("COVID-19"[MeSH Terms] OR "SARS-CoV-2" OR"coronavirus disease 2019" OR "influenza"[MeSH Terms] OR "influenza A virus"[MeSH Terms] OR "influenza B virus"[MeSH Terms] OR "influenza A" OR "influenza B"OR "influenza, human"[MeSH Terms] OR "respiratory syncytial virus"[MeSH Terms] OR "RSV" OR "respiratory syncytial virus" AND
("bacterial coinfection"OR "bacterial co-infection" OR "secondary bacterial infection" OR "bacterial superinfection" OR "antibiotic use" OR "antibiotic prescribing" OR "empirical antibiotic" OR "empiric antibiotic" OR "antimicrobial resistance"[MeSH Terms] OR "drug resistance, bacterial"[MeSH Terms] OR "multidrug resistant" OR "multidrug-resistant" OR "MDR" AND ("hospitalized" OR "hospitalised" OR "hospitalization"[MeSH Terms] OR "inpatient" OR "hospital admission" AND
("adult"[MeSH Terms] OR "adults" OR "middle aged"[MeSH Terms] OR "aged"[MeSH Terms]))

Database 2: Embase

Platform: Embase (via Elsevier; https://www.embase.com)

('COVID-19'/exp OR 'severe acute respiratory syndrome coronavirus 2'/exp OR 'SARS-CoV-2' OR 'coronavirus disease 2019' OR 'influenza'/exp OR 'influenza A' OR 'influenza B' OR 'human influenza'/exp OR 'respiratory syncytial virus'/exp OR 'RSV' OR 'respiratory syncytial virus'
AND ('bacterial infection'/exp OR 'coinfection'/exp OR 'bacterial coinfection' OR 'bacterial co-infection' OR 'secondary bacterial infection' OR 'bacterial superinfection' OR 'antibiotic agent'/exp OR 'antibiotic use' OR 'antibiotic prescribing' OR 'empirical antibiotic' OR 'antimicrobial resistance'/exp OR 'drug resistance'/exp OR 'multidrug resistance'/exp OR 'multidrug-resistant')
AND ('hospitalization'/exp OR 'inpatient'/exp OR 'hospitalized' OR 'hospitalised' OR 'hospital admission')
AND ('adult'/exp OR 'adults' OR 'elderly')

Database 3: Cochrane Central Register of Controlled Trials (CENTRAL)

Platform: Cochrane Library (https://www.cochranelibrary.com)

("COVID-19" OR "SARS-CoV-2" OR "coronavirus disease 2019" OR "influenza" OR "influenza A" OR "influenza B" OR "respiratory syncytial virus" OR "RSV")
AND
("bacterial coinfection" OR "bacterial co-infection" OR "secondary bacterial infection" OR "bacterial superinfection" OR "antibiotic use" OR "antibiotic prescribing" OR "empirical antibiotic" OR "antimicrobial resistance" OR "multidrug resistant" OR "MDR" OR "carbapenem resistant" OR "MRSA")
AND
("hospitalized" OR "hospitalised" OR "inpatient" OR "hospital admission")

Database 4: Web of Science

Platform: Web of Science Core Collection (https://www.webofscience.com)

TS=("COVID-19" OR "SARS-CoV-2" OR "coronavirus disease 2019" OR "influenza A" OR "influenza B" OR "human influenza" OR "respiratory syncytial virus" OR "RSV")
AND
TS=("bacterial coinfection" OR "bacterial co-infection" OR "secondary bacterial infection" OR "bacterial superinfection" OR "antibiotic use" OR "antibiotic prescribing" OR "empirical antibiotic" OR "empiric antibiotic" OR "antimicrobial resistance" OR "multidrug-resistant" OR "MDR" OR "MRSA" OR "carbapenem resistant")
AND
TS=("hospitalized" OR "hospitalised" OR "inpatient" OR "hospital admission")

3. Manual Search and Citation Mining

To ensure completeness of the evidence base, the following supplementary search methods were applied:

- Backward citation tracking: Reference lists of all included studies were manually screened to identify additional eligible records not captured in the database searches.

4. Study Selection Process (PICOS Framework)

In accordance with PRISMA 2020 guidelines, studies were selected based on the following criteria:

- **Population:** Adult patients (≥18 years) hospitalized with laboratory-confirmed COVID-19, influenza A or B, or respiratory syncytial virus (RSV) infection, confirmed by RT-PCR or validated antigen testing.
- **Intervention/Exposure:** Empirical antibiotic use, confirmed bacterial co-infection or secondary bacterial infection, antimicrobial resistance patterns, or associated clinical outcomes.
- **Comparator:** No antibiotic exposure; absence of confirmed bacterial co-infection; or comparator groups as specified in individual studies.

- **Outcomes:** Prevalence of antibiotic use; prevalence of confirmed bacterial co-infection or secondary infection; antimicrobial resistance patterns and organisms; clinical outcomes including mortality, ICU admission, mechanical ventilation, and length of hospital stay.
- **Study Design:** Prospective and retrospective cohort studies, analytical cross-sectional studies, case series, and randomized controlled clinical trials.

5. Management of Exclusions

Studies were excluded from the final synthesis if they:

- Enrolled exclusively paediatric populations (aged <18 years), unless adult-specific data were separately extractable or adults represented ≥80% of participants.
- Did not report at least one of the prespecified outcomes (antibiotic use prevalence, bacterial co-infection prevalence, AMR patterns, or associated clinical outcomes).
- Were systematic reviews, case reports, editorials, commentaries, or conference abstracts without full-text primary data.
- Unpublished or non-peer-reviewed literature, including preprints, dissertations, theses, institutional reports, and other grey literature sources, was not eligible for inclusion.
- Did not include laboratory confirmation of the viral pathogen (RT-PCR or validated antigen testing).
- Reported data from mixed cohorts without virus-specific data extractable by pathogen (n = 3 excluded).
- Did not report adult data separately from paediatric patients and adults represented <80% of the cohort (n = 2 excluded).
- Represented a duplicate cohort with fewer data than a retained publication from the same dataset (n = 1 excluded).

6. Data Synthesis and Statistical Analysis

- **Analysis Model:** Random-effects meta-analysis (DerSimonian-Laird variance estimator) was used to pool prevalence estimates for reported antibiotic use and confirmed bacterial co-infection.
- **Transformation:** The Freeman-Tukey double-arcsine transformation was applied prior to pooling to stabilize variance of proportion data; back-transformed estimates are reported with 95% confidence intervals (CIs).
- **Heterogeneity:** Between-study heterogeneity was quantified using the I2 statistic and Cochran's Q test (I2 <25% = low; 25-50% = moderate; 50-75% = substantial; >75% = considerable).
- **Subgroup Analyses:** Prespecified subgroup analyses were conducted by viral pathogen (COVID-19, influenza A/B, RSV) as the primary reporting unit.

- **Sensitivity Analyses:** Prespecified sensitivity analyses were performed by restricting each subgroup to studies rated as moderate methodological concern using Joanna Briggs Institute (JBI) critical appraisal tools.
- **Publication Bias:** Assessed using Egger's test and Begg's test for subgroups with 10 or more contributing studies. No formal assessment was performed for subgroups with fewer than ten studies.
- **Software:** All statistical analyses were performed using MedCalc Statistical Software version 23.5.5.
