# Supplementary figures and images for: Antibiotic Use, Bacterial Co-Infection, and Antimicrobial Resistance in Adults Hospitalized with COVID-19, Influenza, or RSV: A Systematic Review and Meta-Analysis
Source: Antibiotics (Basel). 2026 Jun 30;15(7):654. doi: 10.3390/antibiotics15070654 (PMC13406035; doi:10.3390/antibiotics15070654)

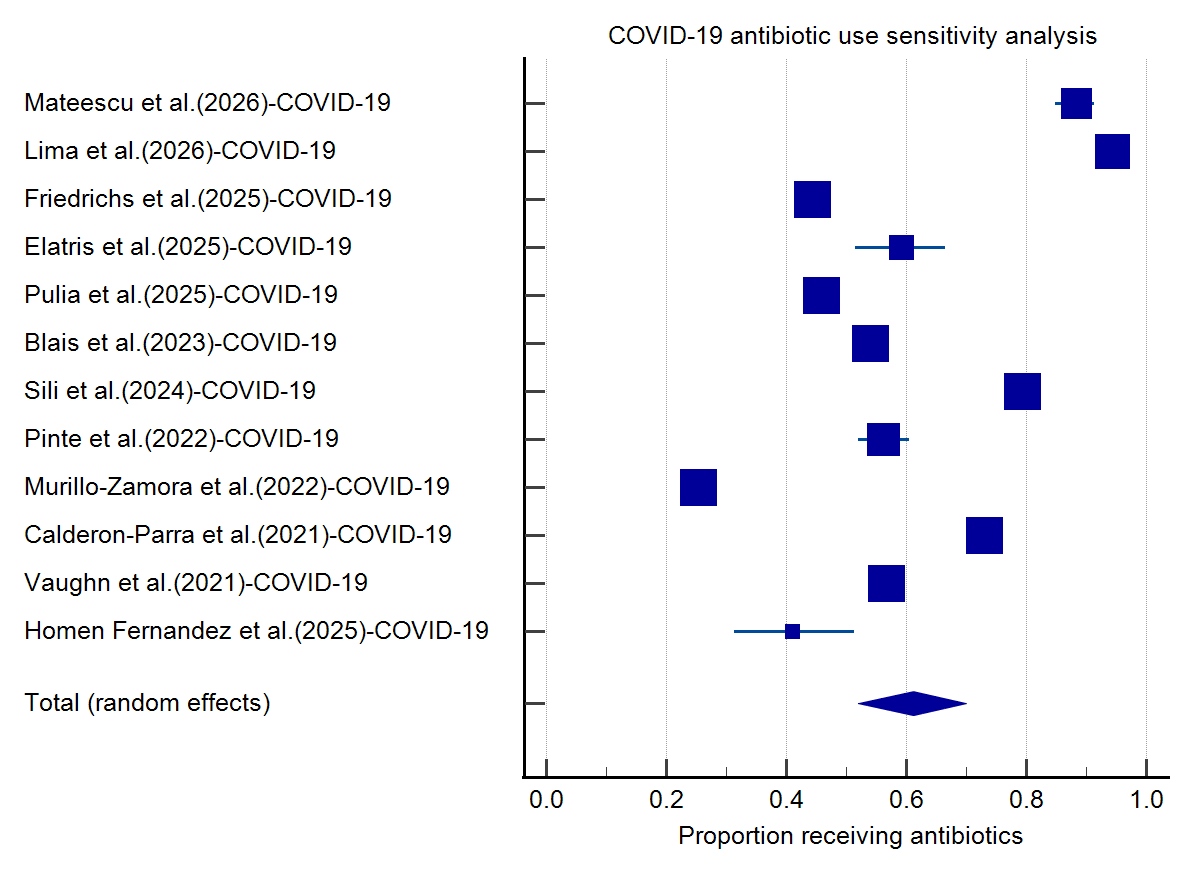

Supplement: Supplementary file 1 [file antibiotics-15-00654-s001.zip › Supplementary figure S1 Covid-19.png]

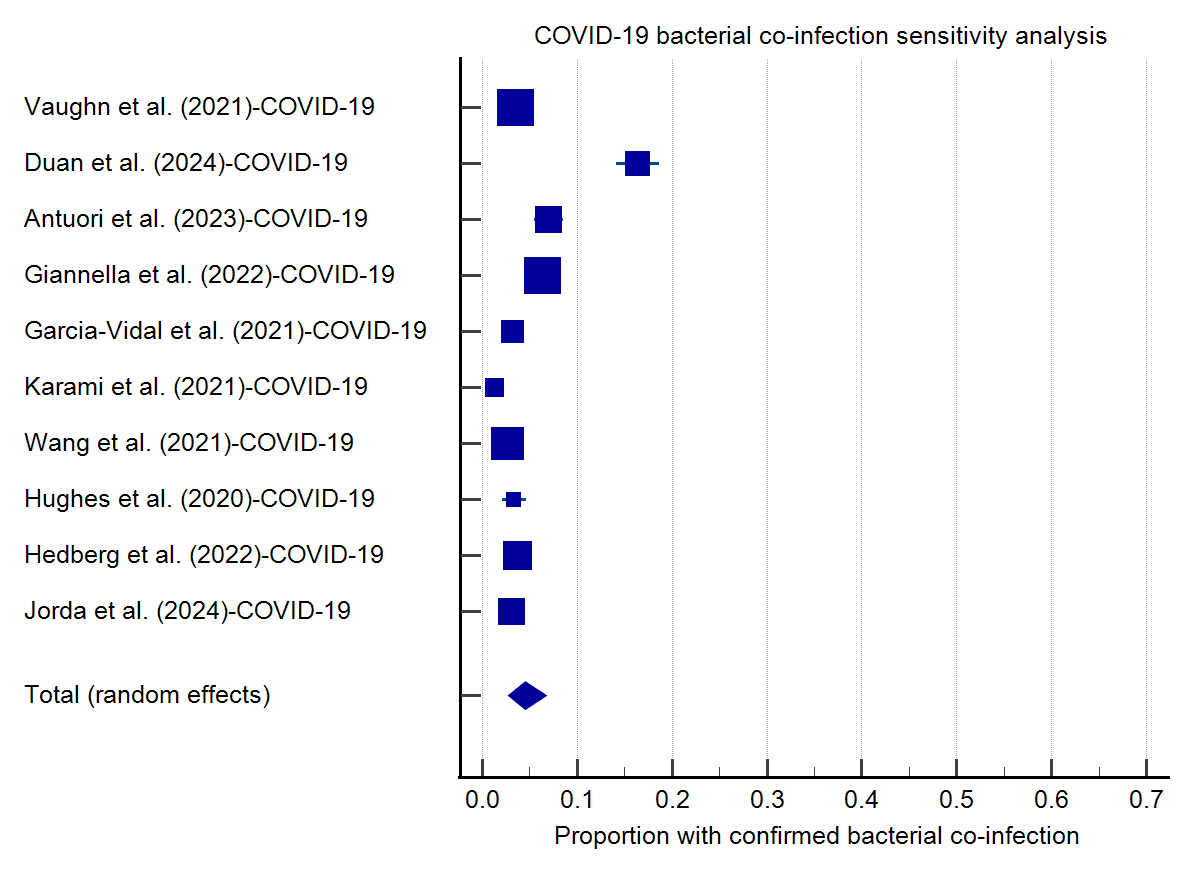

Supplement: Supplementary file 1 [file antibiotics-15-00654-s001.zip › Supplementary figure S2 covid-19.png]
